# Molecular Dynamics Simulations of Graphene and Graphene-Derived Ionic Liquid Supercapacitors: A Review of Methods and Applications

**DOI:** 10.1021/acsmaterialsau.6c00195

**Published:** 2026-10-17

**Authors:** Lucas de S. Silva, Guilherme Colherinhas

**Affiliations:** Instituto de Física, 67824 Universidade Federal de Goiás, Goiânia, Goiás 74690-900, Brazil

**Keywords:** molecular dynamics, ionic liquids, graphene, supercapacitors, electric double layer, electrode-charging methods, quantum capacitance, nanoconfinement, electrode curvature, electrolyte polarizability

## Abstract

Graphene and graphene-derived electrodes combined with ionic liquid electrolytes provide atomically well-defined, high-surface-area interfaces and wide electrochemical windows, making them benchmark platforms for supercapacitor research. Understanding how molecular-scale structure at this interface controls macroscopic capacitance requires molecular dynamics simulation since neither continuum theory nor experiment alone resolves the relevant length and time scales. This review treats the electrode-charging methods available—fixed and constant-charge, constant potential, electronic-structure-aware variants, and applied fields—together with force-field choice, as a toolbox of complementary tools suited to different questions, not a hierarchy of accuracy. We use this framework to organize findings on interfacial structure, electrode curvature, defects, doping, and nanoconfinement, emphasizing how these factors affect both the electric double layer and quantum capacitance. We conclude that progress toward predictive simulation will depend less on selecting a single optimal method than on integrating electrolyte polarizability, realistic electrode electronic response, structural complexity, and transferable machine-learning potentials within experimentally validated frameworks.

## Graphical abstract

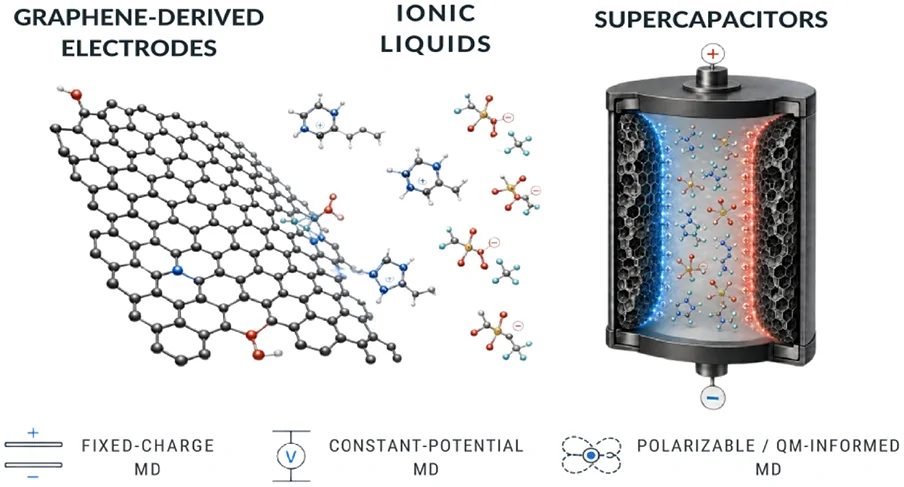

## 1 Introduction

As the transition toward renewable and intermittent energy sources has accelerated over the past two decades, efficient electrical energy storage has become a central technological challenge.^1^ Supercapacitors and batteries have emerged as the two leading electrochemical solutions for this task, and each occupies a distinct region of the power-density–energy-density trade-off. Batteries store energy through redox reactions that transfer electrons between the electrode and the electrolyte, whereas supercapacitors, and in particular electric double-layer capacitors (EDLCs), store charge electrostatically at the electrode–electrolyte interface, without an accompanying charge-transfer reaction. This electrostatic storage mechanism gives EDLCs two key advantages (rapid charge and discharge and long cycle life) but generally results in lower energy density than that of batteries.^1,2^

Closing this remaining energy-density gap depends strongly on the choice of electrode and electrolyte materials. Graphene, first isolated in 2004,^3^ combines an ideally two-dimensional structure, high electrical conductivity, and a large theoretical surface area. It has consequently become one of the most widely studied electrode materials for EDLCs.^4^ Room-temperature ionic liquids (ILs), for their part, offer an electrochemical window several times wider than conventional aqueous electrolytes. Because stored energy scales as *E* ∝ *CV* ^2^, this wider window directly increases the energy density of the device, and ILs have accordingly found application across a broad range of energy storage and conversion technologies.^5,6^ The combination of a graphene electrode with an IL electrolyte therefore represents one of the most attractive material platforms currently available for next-generation supercapacitors.

Realizing this potential requires a molecular-level understanding of how the structure of the IL at the graphene surface controls the interfacial or differential capacitance, and this is not a problem that classical electrochemical theory can solve on its own. The Helmholtz model^7^ and its later refinements by Gouy, Chapman, and Stern^8–10^ describe the electric double layer as a dilute system in which ions interact with the electrode surface but not significantly with one another. A neat ionic liquid violates this assumption completely since, every ion is, by definition, surrounded by other ions at all times, with no solvent to screen the interactions between them.

This fundamental difference motivated a shift from dilute-solution electrochemistry toward statistical-mechanical descriptions of dense Coulomb fluids. Kornyshev argued in 2007 that this called for a genuine paradigm change in double-layer theory, replacing dilute-solution electrochemistry with the statistical mechanics of dense Coulomb fluids,^11^ and an independent treatment based on modified Poisson–Nernst–Planck equations reached a compatible conclusion around the same time.^12^ The mean-field theories that followed this shift predicted qualitatively new behavior, including a crossover from an overscreened, oscillatory charge-density profile at low potential to a crowded, saturated regime at high potential.^13,14^ This crossover can produce camel- or bell-shaped differential capacitance curves, which may also exhibit pronounced asymmetry.^15^ Later refinements incorporated short-range ion correlations^16^ and explicit interfacial layering,^17^ and in every case these theoretical advances were developed alongside and validated against molecular-resolution simulations.

A striking experimental confirmation of how far ionic liquids depart from classical double-layer behavior was reported by Largeot et al.^18^ in 2008. Using a carbide-derived carbon electrode in a neat imidazolium ionic liquid, the authors found that the capacitance was maximized rather than suppressed when the electrode pore size was reduced to approximately match the size of the bare ion. This result directly contradicts the classical expectation that pores narrower than a solvated ion should be electrochemically inactive. Explaining this anomalous increase in capacitance required an ion-resolved description that could be accessed through molecular simulation. Subsequent molecular dynamics studies of nanoconfined ionic liquids identified partial ion desolvation and a superionic charge-storage state as the underlying mechanism^19^ and later work showed that the capacitance oscillates rather than decreasing monotonically as the pore size is reduced further.^20^ This case illustrates how molecular simulations can resolve experimental observations that are not readily explained by continuum models.

Graphene occupies a distinctive position within the broader body of work. Unlike the disordered, heterogeneous pore networks of activated or carbide-derived carbons, a graphene electrode is atomically flat, chemically homogeneous, and structurally well-defined, which makes it a natural model system in which to isolate the effect of curvature, edge defects, or heteroatom doping, one variable at a time (Figure 1).

**Figure 1:**
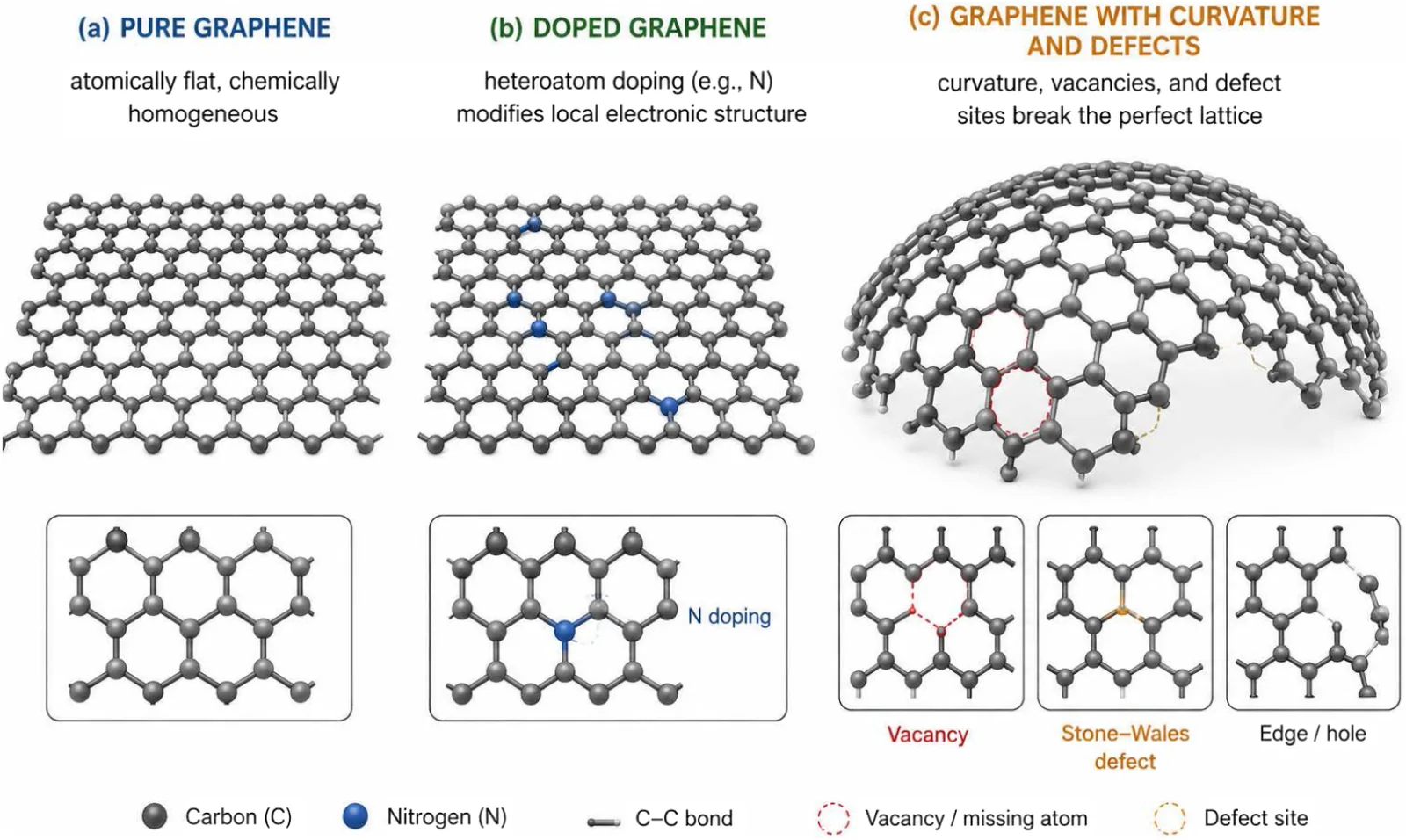
Representative graphene model systems used in molecular dynamics simulations of supercapacitors: (a) pristine graphene, (b) heteroatom-doped graphene (illustrated by nitrogen substitution), and (c) graphene with curvature and representative structural defects (vacancies, Stone–Wales defects, and edge defects). These model structures enable the systematic investigation of how surface chemistry, topology, and electronic structure influence ion adsorption, electric double-layer formation, and quantum capacitance.

Direct experimental probes of graphene and graphite in contact with ionic liquids, using capacitance measurements^21^ and interfacial force spectroscopy,^22–24^ confirm that the layered, oscillatory ion-density profiles predicted by theory persist at this idealized electrode, consistent with the structure–capacitance relationships extracted from atomistic simulations.^25^ At the same time, graphene’s semimetallic band structure introduces a complication that most porous carbon electrodes allow simulators to ignore. Because the density of states near the Fermi level is finite rather than divergent, the electrode itself contributes a non-negligible quantum capacitance^26^ in series with the double-layer capacitance of the electrolyte. This quantum contribution depends sensitively on the same structural features used to tune the double-layer response, including curvature^27^ and heteroatom doping;^28^ capturing both contributions simultaneously, with comparable accuracy, remains a genuinely open methodological problem. This problem is a central thread of the present review.

Several reviews already cover part of this broader field. Bo et al.^29^ reviewed molecular dynamics studies of supercapacitor electrode design across porous, nanostructured, and planar graphene geometries, with an emphasis on electrode engineering rather than the electrolyte side of the interface. Jeanmairet, Rotenberg, and Salanne provide a comprehensive treatment of microscopic simulation methods for electrochemical double-layer capacitors across electrode materials in general.^30^ Wu reviews the double-layer structure, capacitance, and charging dynamics from a statistical-mechanical perspective.^31^ Zhang et al. focus on the theory and simulation of ionic liquids under nanoconfinement.^32^ Most recently, Nesterova et al.^33^ critically reviewed how surface material properties shape nanoconfined ionic liquids across a range of electrode chemistries. None of these reviews are organized specifically around graphene, and none could incorporate the methodological developments of the past two to three years, including electronic-structure-aware constant-potential methods and the first machine-learning interatomic potentials applicable to charged ionic-liquid interfaces.

The present review therefore adopts a narrower material focus in exchange for closer engagement with these recent developments. Its methodological sections are organized explicitly around the idea of complementarity: the electrode-charging methods, force fields, and modeling strategies available to the simulator each answer a different physical question well, and none should be treated as a general-purpose replacement for the others. Section 2 presents the toolbox of electrode-charging methods and, in Section 2.6, compares all four side by side; Section 2.5 addresses the choice of force field. Sections 3.1–3.3 apply this combined toolbox to interfacial structure, electrode engineering, and nanoconfinement, and Section 3.4 discusses the emerging role of machine-learned interatomic potentials. Section 4 lays out the challenges that remain, and Sections 5 and 6 close with a summary, outlook, and concluding remarks.

## 2 Simulation Methods for Graphene/Ionic-Liquid Supercapacitors

Any molecular dynamics simulation of a supercapacitor must specify how the electrode responds electrostatically to the instantaneous configuration of the electrolyte. Four broad strategies are currently used to solve this problem: assigning the electrode a fixed charge distribution, letting the electrode charge fluctuate to maintain a constant potential, extending either approach to account explicitly for the electrode’s electronic structure, or applying an external field directly, without assigning charges to individual electrode atoms at all. This section reviews each of these strategies in turn and argues that they are best regarded as a toolbox suited to different physical questions rather than a hierarchy of increasing accuracy.

### 2.1 Fixed and Constant-Charge Methods

In the fixed-charge method (FCM), also termed the constant-charge method (CCM), each electrode atom is assigned a partial charge, typically to reproduce a target surface charge density, and is held constant for the remainder of the simulation.^34^ The electrode therefore exerts an electrostatic influence on the electrolyte, but does not respond to it. Because no additional electrostatic solve is required at each time step, FCM is computationally inexpensive, and it is a well-established choice for studies focused on structural or equilibrium properties, such as the layering of ions near a surface or how that layering changes across a series of electrolytes or electrode geometries.^35^

The main limitation of FCM concerns the electrode’s dynamical response. Because the charge distribution is imposed rather than computed self-consistently, FCM cannot represent the polarization of the electrode by the electrolyte. Comparisons against constant-potential simulations show that this omission affects both the fine structure of the adsorbed ion layer and, more substantially, the electrode’s behavior during charging and discharging transients.^34^

### 2.2 The Constant-Potential Method

The constant-potential method (CPM) treats the electrode as an ideal conductor. Charges on the electrode atoms are allowed to fluctuate at every time step, adjusted through a variational or matrix-inversion procedure so that the entire electrode surface remains at a single externally imposed potential.^36,37^ The method was first developed for planar water/electrode systems and was later extended to ionic liquids at metallic electrodes.^36,37^ CPM is the standard choice whenever the electrode’s dynamical response is itself of interest (such as charging and discharging kinetics) or when the electrode geometry is heterogeneous, curved, or defective, conditions under which a uniform fixed charge is a poor approximation of a real conductor.^38^

The added computational cost of updating electrode charges at every time step has historically limited the use of CPM for large systems or long trajectories. Tee and Searles showed that fully periodic implementations, which avoid the vacuum-slab geometry traditionally required for constant-potential simulations, substantially reduce this overhead, and can, in some cases, make CPM cheaper than the fixed-charge alternative for an equivalent system.^39^ A related development constrains the electrode’s total charge rather than its potential while still allowing the charge to redistribute dynamically among the electrode atoms to satisfy the equipotential condition. Tee and Searles demonstrated that this constrained-charge ensemble is thermodynamically conjugate to the standard constant-potential ensemble, recovering the same equilibrium averages while differing in fluctuations and computational convenience.^40^

Even proponents of CPM note that it is not a universal replacement for the FCM. Tee and Searles observed that the added complexity of updating electrode charges at every time step is not justified whenever the property being extracted does not depend closely on the fine details of the electrode charge distribution.^39^ The choice between FCM and CPM is therefore a question of which physical quantity is being extracted and not a question of which method is more accurate in general.

### 2.3 Electronic-Structure-Aware Variants

The standard CPM treats the electrode as an ideal, structureless conductor. This assumption does not hold for graphite and graphene, whose densities of states near the Fermi level are finite rather than divergent.^41^ As a result, the electrode itself contributes a non-negligible quantum capacitance in series with the double-layer capacitance of the electrolyte; because the total capacitance of components connected in series is dominated by the smaller contribution, the standard CPM, which implicitly assumes an infinite quantum capacitance, systematically overestimates the total capacitance measured experimentally for these materials, especially near the potential of zero charge, where the true quantum contribution is smallest.^42^

A conceptually distinct, electronic-structure-aware strategy is the quantum mechanics/molecular dynamics (QMMD) approach developed by Elliott and coworkers.^43,44^ Unlike conventional CPM, which adjusts electrode charges to maintain a prescribed potential, QMMD constrains the total charge of the graphene electrode while allowing the atomic charge distribution to evolve in response to the instantaneous electrolyte configuration. In the implementation of Elliott et al., the electrolyte is represented by classical point charges, which generate the electrostatic environment for a self-consistent-charge density-functional tight-binding (SCC-DFTB) calculation of the graphene electrode. Mulliken charges obtained from the electronic-structure calculation are then used to update the partial charges of the electrode atoms before the classical MD trajectory is continued.

This iterative coupling introduces a dynamically polarizable, nonlocal electrode response without requiring a full first-principles description of the electrolyte at every MD time step. Elliott et al.^44^ used this strategy for graphene in contact with aqueous LiCl, NaCl, KCl, MgCl_2_, and CaCl_2_ electrolytes and performed simulations extending to tens of nanoseconds, with the electronic structure updated periodically during the trajectory. The method therefore occupies an intermediate regime between fully classical polarizable models and first-principles molecular dynamics: it retains an explicit quantum-mechanical description of charge redistribution within the electrode while preserving much of the length- and time-scale accessibility of classical MD.

The approach also has its important limitations. Only the electrode is treated as dynamically polarizable, whereas the solvent and ions retain the fixed partial charges of their classical force fields. Consequently, short-range charge-transfer interactions between the electrolyte and the surface are not described explicitly, and the accuracy of the method still depends on the classical electrolyte model and the frequency with which the quantum-mechanical charge redistribution is updated. QMMD should therefore be regarded as a hybrid multiscale strategy rather than as a complete replacement for fully quantum-mechanical dynamics.

Goloviznina et al.^42^ addressed this limitation by parametrizing the metallicity of the electrode directly from a quantum chemistry calculation of its density of states. Using this approach, the authors reproduced the experimentally observed capacitance minimum near the potential of zero charge for graphite in contact with both aqueous and ionic-liquid electrolytes. In a related study, Wang et al.^45^ incorporated electron spillover, the tendency of the electrode’s electron density to extend beyond its formal geometric boundary, directly into the constant-potential formalism, again recovering capacitance behavior in closer agreement with experiment. For electrodes that combine multiple elements with different electronegativities, such as MXenes, Lin et al.^46^ extended the same logic to a heteroatomic constant potential method (HCPM), fitted to density-functional-theory partial charges to represent element-specific charge response. These extensions are particularly relevant to graphene, a homogeneous, single-element surface whose only microscopic degrees of freedom—curvature, defects, and doping (Section 3.2)—act simultaneously on the double-layer and quantum-capacitance contributions.

### 2.4 Externally Applied Field Methods

A fourth family of methods departs from the electrode charge picture entirely. Instead of distributing charge over electrode atoms, finite-field methods impose a macroscopic electric field *E⃗*, or fix the electric displacement *D⃗*, directly through the simulation Hamiltonian, following the modern theory of polarization.^47,48^ Zhang and Sprik^49,50^ adapted this framework to classical molecular dynamics of charged solid/electrolyte interfaces, showing how to recover the capacitance of an insulator/electrolyte double layer under either fixed-*E⃗* or fixed-*D⃗* boundary conditions. Because this approach does not require the surface to be a conductor, it is well suited to systems that the standard CPM cannot easily treat, such as electrolytes in contact with an insulating or semiconducting phase.

Externally applied fields are not mutually exclusive to constant-potential electrodes. Dufils et al.^51^ combined a finite field with a single constant-potential electrode, generating two independent electrochemical interfaces from opposite faces of the same metal slab, and demonstrated that the two control strategies can be used together rather than treated as alternatives. Bonnet et al.^52^ proposed a related first-principles strategy in which the electrode is held at a constant electron chemical potential rather than at a constant field.

Section 2.6 compares all four electrode-charging strategies introduced in this section side by side. The electrode-charging method, however, is only one of two methodological choices that determine the quantitative reliability of a graphene-ionic liquid simulation. The other, the force field used to describe the ionic liquid itself, is discussed in the next section.

### 2.5 Force Fields for Ionic Liquids

The electrode-charging method discussed in Section 2 determines how the simulation responds to the electrolyte’s instantaneous configuration. The force field determines whether that configuration is itself realistic. The two choices are formally separable at the implementation level but physically coupled in determining the accuracy of the interfacial description.

The default choice for both cations and anions remains a nonpolarizable, atomistic force field with fixed partial charges, typically derived from the Canongia Lopes–Pádua (CL and P) family^53^ or related OPLS-AA-compatible parametrizations.^54^ These models remain the practical choice when large systems or long trajectories are required (for example, in nanoconfinement studies, electrolyte screening, or charging simulations) because more sophisticated treatments may be computationally prohibitive.

Yan et al.^55^ showed that when ions carry their full formal integer charge, these models underestimate self-diffusion and ionic conductivity while overestimating viscosity because they omit the charge-transfer and electronic screening effects that soften the effective electrostatic interaction between ions in a real ionic liquid. A widely used correction, scaling all partial charges uniformly to some fraction of their formal value, typically between 0.7 and 0.8, brings the simulated dynamics substantially closer to experiment at negligible additional computational cost.^56^ However, the appropriate scaling factor is not transferable between different cation–anion combinations and must be retuned for each new electrolyte.^57^ As with the choice between FCM and CPM in Section 2, whether this trade-off is worth making depends on the property being extracted: static structure and equilibrium capacitance are far less sensitive to it than charging kinetics or diffusion-limited processes. Explicitly including electronic polarizability, most often through Drude oscillators or induced point dipoles, addresses the dynamics problem at its source rather than through empirical rescaling. Yan et al.^55^ demonstrated that polarizability substantially improves agreement with experimental shear viscosity and diffusion coefficients in imidazolium ionic liquids. Borodin^58^ subsequently developed polarizable force fields across a wide range of cation and anion chemistries, and Goloviznina et al.^59^ introduced the CL and Pol force field, a transferable, general-purpose polarizable model compatible with the widely used CL and P parameter set, using Drude-induced dipoles fitted to first-principles dispersion and induction calculations rather than to bulk experimental properties. A comprehensive review of the resulting landscape of polarizable models for ionic liquids and related electrolytes is given by Bedrov et al.^57^

For graphene and graphite electrodes, polarizability matters on both sides of the interface simultaneously. The electronic response of the electrode is captured by the constant-potential methods discussed in Section 2, but the electronic response of the electrolyte to the electrode’s field is a separate physical effect, governed by the force field alone. Frömbgen et al.^60^ derived polarizable force-field parameters explicitly for an ionic liquid/graphite interface, rather than relying on standard combining rules between separately parametrized liquid and solid force fields, and found that this explicit parametrization measurably changed the structure of the adsorbed layer. This finding is corroborated from the electrode-charging side: Bacon et al.^61^ showed that quantitative agreement with experimental capacitance in a nanoporous carbon/ionic-liquid supercapacitor was obtained only once mutual polarization of both the electrode and the electrolyte was included together, with either one alone proving insufficient. Force-field polarizability and electrode-charging method are therefore not independent choices to be optimized separately, but two components of the same physical description of the interface. Coarse-grained representations, which collapse each ion to as few as one or three interaction sites, sacrifice atomistic detail in exchange for the larger system sizes and longer trajectories required by charging-dynamics studies (Section 2.2) and nanoconfinement studies (Section 3.3). Tee and Searles used a coarse-grained [BMIM]­[PF_6_] model throughout the constant-potential method comparisons discussed in Section 2, retaining the qualitative electric double-layer structure and capacitance of the system while keeping the cost low enough to run the many independent simulations needed to compare electrode-charging methods against one another.^39^ This trade-off interacts with the electrode-charging axis in a specific way: coarse-graining is most defensible exactly when the quantity of interest is insensitive to atomistic detail at the interface, the same condition under which Section 2.2 identified fixed-charge electrodes as an adequate and lower-cost choice. The conditions that justify coarse-graining often overlap with those that justify a fixed-charge electrode, although the validity of each approximation should be assessed independently.

The rapid recent growth of this literature is itself worth noting (Figure 2). Recent developments further show that force-field fidelity depends not only on the description of the electrolyte but also on how the electrode response is represented. Ye and Wang^62^ developed a coarse-grained model of room-temperature ionic liquids confined between metallic electrodes to test predictions derived from mean-field theories. Their simulations reproduced spontaneous surface-charge separation and revealed enhanced in-plane ionic ordering at the interface. More importantly, comparison between perfectly conducting and nonmetallic electrodes showed that image-charge interactions substantially increase the differential capacitance and the predicted energy-storage capability. These results expose an important limitation of coarse-grained models that neglect electrode polarization. A distinct data-driven direction has emerged from the studies of Naeiji et al.^63^ and Ajide et al.,^64^ who combined on-the-fly machine-learned force fields with electronic-structure calculations to investigate pristine, vacancy-containing, and N-, O-, or S-doped HOPG, as well as selected HOPG–water and graphene-nanoribbon interfaces. Their results demonstrate that defects and dopants can introduce localized or midgap states, shift the Fermi level, modify orbital hybridization, and alter the structure of interfacial water. Because these studies do not explicitly model a complete ionic-liquid supercapacitor, however, their conclusions are better regarded as mechanistic guidance for electrode design than as direct predictions of device capacitance. Di Pasquale et al.^65^ addressed a complementary problem by training a multioutput neural network to reproduce the redistribution of electronic charge within a graphene electrode in contact with an aqueous electrolyte over different surface-charge states. The model agreed closely with quantum-mechanical molecular dynamics, while allowing the polarization response to be incorporated into classical simulations at substantially lower cost. Collectively, these approaches target different sources of force-field error (image forces at metallic interfaces, the atomistic energetics of defective carbon electrodes, and dynamic charge redistribution), but their integration into a transferable model for graphene/ionic-liquid supercapacitors remains an open challenge.

**Figure 2:**
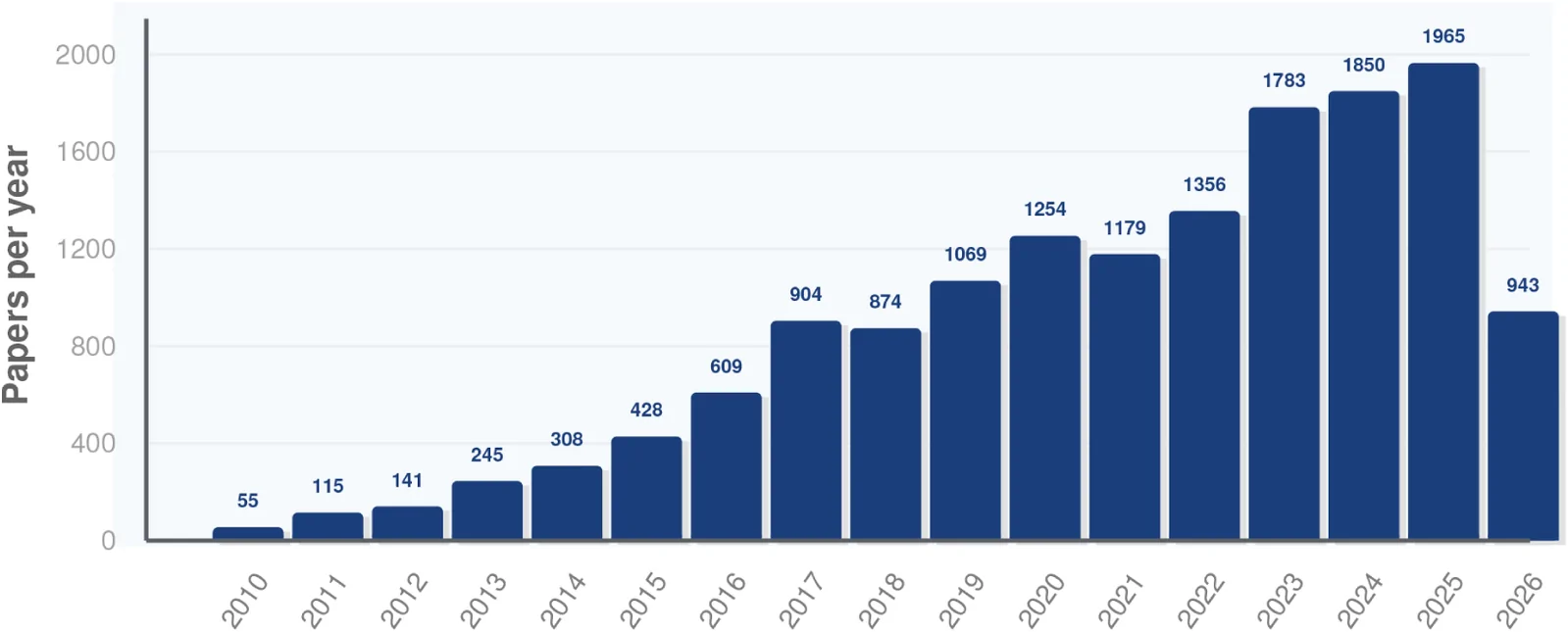
Web of Science total result counts for the topic search (electric double layer capacitors OR supercapacitors OR EDLC) AND molecular dynamics AND graphene electrodes, 2010–2026 (2026 partial, through July). This reflects the broader growth of computational research at the intersection of these topics rather than a narrow count of graphene-electrode MD studies specifically.

Force field choice and electrode-charging method therefore define a two-dimensional design space—nonpolarizable to fully polarizable on one axis, fixed-charge to fully electronic-structure-aware on the other—that the field has only recently begun to explore systematically. Machine-learning interatomic potentials, discussed in Section 3.4, do not add a third axis to this space so much as attempt to collapse it, reproducing the accuracy of the polarizable, electronic-structure-aware corner at a computational cost closer to the nonpolarizable, fixed-charge one.

The comparison among fixed-charge, charge-scaled, polarizable, and coarse-grained force fields also requires more nuance than their nominal level of physical detail may suggest. Charge scaling can improve transport properties while simultaneously modifying ion–electrode electrostatics, whereas explicitly polarizable models may reproduce bulk dynamics more accurately without necessarily guaranteeing an equally accurate description of short-range adsorption at graphene. Likewise, a force field validated for density, viscosity, or self-diffusion in the bulk is not automatically transferable to a strongly charged or nanoconfined interface. The classification of a model as polarizable or nonpolarizable should therefore not be treated as a sufficient indicator of accuracy. Meaningful assessment requires validation of both bulk and interfacial properties, explicit reporting of charge-scaling factors and cross-interaction parameters, and sensitivity analyses showing whether the conclusions persist across reasonable parameter choices.

### 2.6 Comparison between Electrode-Charging Methods

The four-electrode charging strategies discussed in this section (FCM, CPM, electronic-structure-aware CPM, and externally applied fields) are not competing solutions to the same problem. Each is built around a different physical quantity being held fixed, and each is therefore best suited for a different class of question. Beyond these conceptual differences, however, the methods also differ in computational overhead, convergence requirements, accessible system sizes and time scales, and practical limitations. Table 1 summarizes this comparison directly, with the electronic-structure-aware family split into DOS-aware CPM and QMMD columns, so that the methods can be read side by side rather than argued for individually.

**Table 1:** Practical Comparison of Complementary Electrode-Charging and Electronic-Response Strategies Relevant to Molecular Dynamics Simulations of Graphene/Ionic-Liquid Supercapacitors^a^

**Criterion**	**Fixed/constant charge (FCM)**	**Constant potential (CPM)**	**DOS-aware/quantum-capacitance CPM**	**QM-informed MD (QMMD)**	**Externally applied field**
Physical constraint	Prescribed charge on each electrode atom; no dynamic local charge response.	Electrode potential is prescribed; atomic charges fluctuate to maintain the equipotential condition.	Electrode potential is prescribed while the charge response incorporates the finite electronic density of states or related quantum-capacitance information.	Total electrode charge is constrained, while its atomic charge distribution is periodically recalculated from the instantaneous electrolyte environment using an electronic-structure method.	A macroscopic electric field *E⃗* or electric displacement *D⃗* is imposed across the simulation cell.
Computational burden	Generally lowest; no self-consistent electrode-charge determination is required during MD.	Additional overhead from repeatedly solving forelectrode charges; efficient implementations can substantially reduce this cost.^39^	Higher complexity than conventional CPM because electronic-structure information must be parametrized or otherwise incorporated into the electrode response.	Higher than classical MD because periodic electronic-structure calculations are required, but substantially more accessible than performing first-principles MD over the entiretrajectory.	Strongly implementation-dependent; imposing thefield itself is inexpensive, whereas the underlying classical orfirst-principles framework determines the overall cost.
Practical system size/time scale	Well suited to large systems, long trajectories, and systematic screening.	Large classical-MD systems and nanosecond trajectories are feasible with modern implementations.	Generally less convenient for extensive screening; practical limits depend on how the electronic response is represented.	Intermediate between classical and first-principles MD; DFTB-based implementations have enabled trajectories extending to tens of nanoseconds.^44^	Ranges from large classical-MD cells to much smaller and shorter first-principles MD simulations.
Convergence/numerical considerations	Sampling, imposed surface charge, finite-size effects, and electrostatic boundary conditions must be assessed.	Requires sufficiently converged electrode charges; behavior depends on the charge solver, electrostatics, geometry,and numerical tolerance.	Requires convergence of the constant-potential response and validation of the electronic-structure-derived description.	Depends on electronic-structure convergence, the interval between QM charge updates, charge-partitioning procedure,and the underlying classical force field.	Requires careful treatment of periodic boundary conditions, field direction, cell geometry, and the relation between the imposed field and interfacial potential difference.
Main applicability limits	Cannot reproduce instantaneous local electro depolarization and may deviate under strong polarization, heterogeneity, or confinement.	Conventional CPM treats the electrode approximately as an ideal metal and does not automatically capture finite-DOS or quantum-capacitance effects.	Accuracy depends on the electronic model or parametrization; greater methodological complexity than conventionalCPM.	In current implementations, the electrolyte mayremain classically described and nonpolarizable; explicit electrode–electrolyte charge transfer is not necessarily captured,and the result depends on the chosen QM description.	The applied field is not itself a microscopic electrode model; additional treatment is required when local electrode-charge response is important.
Best suited for	Structural trends, electrolyte screening, and cases where prescribed surface charge is adequate.	Charging dynamics and heterogeneous, curved, or confined interfaces where local polarization is important.	Graphene, graphite, and other electrodes for which finite density of states and quantum capacitance significantly affect the total response.	Graphene and related electrodes when nonlocal, electrolyte-induced charge redistribution must be described while retaining trajectories beyond routine first-principles MD time scales.	Periodic electrochemical cells, systems containing insulating components, and field-controlled classical or first-principles simulations.
Representative reference	Merlet et al.^34^	Reed et al.^37^	Goloviznina et al.^42^	Elliott et al.^44^	Dufils et al.^51^

a Computational cost and accessible system size are given qualitatively because they depend strongly on implementation, electrostatic solver, hardware, convergence criteria, and force field.

Direct, quantitative comparisons between FCM and CPM illustrate why the practical distinctions summarized in Table 1 matter. Wang et al.^38^ compared the two methods for a LiClO_4_/acetonitrile electrolyte at a graphite electrode and found close agreement at low potential differences (ΔΨ ≤ 2 V), whereas substantial differences emerged at higher voltages. At ΔΨ ≥ 4 V, CPM predicted markedly stronger Li^+^ adsorption because the electrode charge could respond to local fluctuations in the electrolyte. Yang et al.^66^ extended this comparison to nanometer and subnanometer pores and likewise showed that agreement between the two descriptions deteriorates when local polarization and confinement effects become increasingly important. These comparisons demonstrate that the additional computational machinery of CPM becomes most relevant precisely in regimes where the assumption of a spatially fixed electrode charge becomes physically restrictive. Zeng et al.^67^ and the broader review by Zhan et al.^68^ reaffirm this trade-off systematically across the field.

Computational cost should nevertheless not be interpreted as a simple hierarchy among the methods. FCM avoids the repeated solution of electrode charges and is therefore particularly attractive for large systems, long trajectories, and systematic screening. CPM introduces an additional charge-determination step, but modern algorithms and periodic implementations have substantially reduced this overhead, making constant-potential simulations increasingly practical for large classical MD systems.^39^ Electronic-structure-aware approaches introduce a further level of complexity because the electrode response must also reflect its finite electronic density of states, whereas externally applied fields can range from relatively inexpensive classical MD implementations to substantially more demanding first-principles MD calculations. Consequently, the relevant question is not which method is intrinsically “best”, but whether its additional computational cost resolves a physical degree of freedom required for the observable and regime of interest.

None of the columns of Table 1 therefore identifies one approach as universally superior. A computationally efficient method suited to structural screening, such as FCM, is not intrinsically less rigorous than a method designed to resolve potential-dependent electrode polarization, such as CPM; rather, the two descriptions retain different physical degrees of freedom. The same reasoning extends to electronic structure-aware and field-based approaches. Moreover, the electrode charging model constitutes only one component of the simulation protocol and must be considered together with the force field choices discussed in Section 2.5, the electrostatic boundary conditions, system dimensions, and the physical observable being calculated.

Two open-source packages, MetalWalls and ELECTRODE,^69,70^ already implement several of these methods side by side within the same simulation engine, facilitating controlled methodological comparisons. A broader treatment of electrode/electrolyte interface simulation, covering all of the methods compared here, is given by Scalfi et al.^71^ The practical implication for the rest of this Review is that the choice of electrode-charging method used in a given study should be interpreted in relation to the physical question and observable being investigated rather than as an indication of the intrinsic rigor of the work.

At the same time, the existing comparisons do not yet define general quantitative criteria for selecting among these methods. The potential differences or confinement regimes at which FCM and CPM begin to diverge have been established for only a limited number of electrode–electrolyte combinations, and thresholds identified for one system cannot be assumed to apply to another. Method performance depends jointly on electrode geometry, electrolyte composition, force-field treatment, boundary conditions, sampling, and the observable being calculated. Table 1 should therefore be interpreted as a problem-selection guide rather than as a universal decision rule. Systematic benchmark calculations in which the same graphene/ionic-liquid system is simulated using multiple charging ensembles while all other parameters are held fixed remain necessary to quantify the uncertainty introduced specifically by the electrode model.

### 2.7 Practical Limitations and Sources of Uncertainty

Beyond the choice of electrode-charging method and electrolyte force field, the reliability of molecular simulations of electrochemical interfaces depends strongly on the construction of the simulation cell and the extent of configurational sampling. These sources of uncertainty are common to both fixed-charge and constant-potential approaches and can complicate quantitative comparisons even when the underlying electrode model is otherwise appropriate. Figure 3 summarizes the main sources of uncertainty considered in this section.

**Figure 3:**
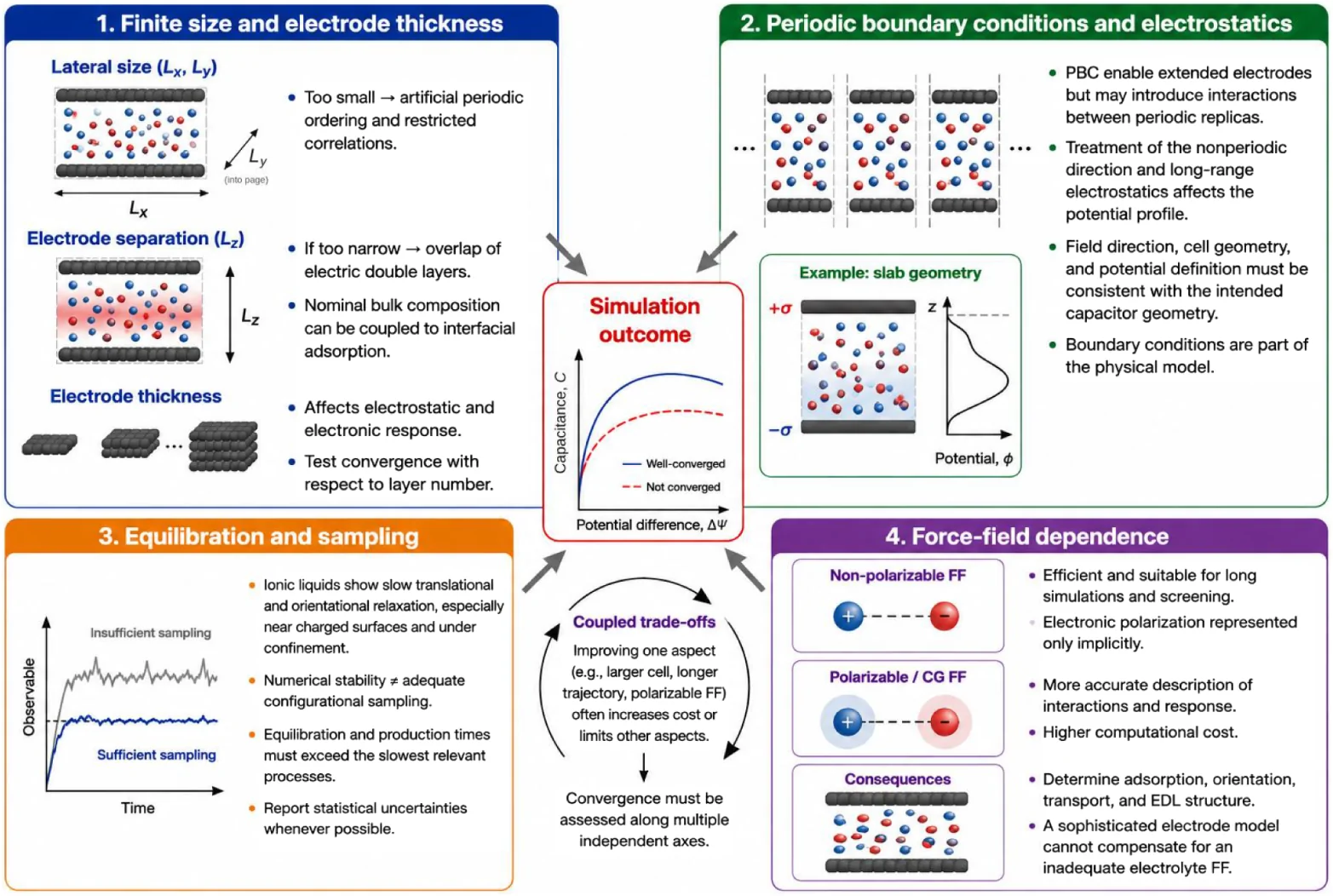
Main sources of uncertainty and practical limitations in molecular dynamics simulations of graphene/ionic liquid supercapacitors. Finite system dimensions, electrode separation and thickness, periodic boundary conditions and electrostatic treatment, equilibration and configurational sampling, and force-field parametrization can independently affect the predicted interfacial structure and electrochemical response. These factors are also interdependent, creating trade-offs between physical fidelity, accessible system size, simulation time scale, and computational cost. Reliable interpretation, therefore, requires convergence to be assessed along multiple methodological axes rather than through a single criterion.

Finite-size effects constitute one of the most fundamental limitations. The lateral dimensions of the electrode must be sufficiently large to accommodate the relevant interfacial correlations without imposing artificial periodic ordering, while the electrode separation must allow the electrolyte to recover bulklike behavior away from the interfaces. If the electrolyte region is too narrow, then the electric double layers associated with opposing electrodes may interact directly, and the nominal bulk composition can become coupled to interfacial adsorption.

A related limitation arises when substantial ion adsorption at the electrode depletes the nominal bulk electrolyte region. In a conventional closed simulation cell with a fixed number of particles, the accumulation of ions within the electric double layer can alter the composition of the remaining bulk-like solution, so that the interface is effectively sampled at a concentration different from the intended reservoir concentration. Constant-chemical-potential molecular dynamics (CμMD) provides a strategy to mitigate this problem by introducing a pseudo-open boundary condition that maintains a target composition in a control region representative of the bulk reservoir. Di Pasquale et al.^72^ combined this approach with QMMD in the CμQMMD framework, simultaneously maintaining an electroneutral bulk solution at the prescribed concentration and accounting for the dynamic redistribution of partial charges within the graphene electrode. This combination illustrates that reliable simulations of strongly adsorbing interfaces may require control of both the electrode electronic response and the electrolyte reservoir composition.

The thickness of the electrode model introduces an additional convergence parameter because an atomically thin graphene sheet, a few-layer graphene electrode, and a semi-infinite graphite-like electrode do not necessarily produce identical electrostatic or electronic responses. Consequently, capacitance and interfacial structure should ideally be tested for convergence with respect to lateral cell dimensions, electrode separation, and electrode thickness rather than assuming that a single simulation cell geometry is representative of the macroscopic interface.

On the other hand, periodic boundary conditions are indispensable for representing extended electrodes with tractable simulation cells, but the resulting electrostatic problem is not equivalent to that of an isolated capacitor unless the cell geometry and long-range electrostatic treatment are chosen consistently. Artificial interactions between periodic replicas, the treatment of the nonperiodic direction, and the definition of the potential difference across the cell can all influence the calculated electrostatic profile. These considerations become particularly important for slab geometries, external applied fields, and strongly polarized interfaces. The appropriate boundary conditions are therefore part of the physical model rather than merely a technical simulation setting.

Sampling provides a distinct source of uncertainty that should not be confused with numerical convergence. Ionic liquids can exhibit slow translational and orientational relaxation, particularly near charged surfaces and under nanoconfinement. The markedly different ionic mobilities reported for chemically distinct ionic liquids, even when their equilibrium capacitances are similar, illustrate why comparable trajectory lengths do not necessarily provide comparable statistical sampling. A simulation may therefore achieve stable temperature, pressure, and energy and, in CPM, well-converged instantaneous electrode charges while still failing to sufficiently sample the exchange of ions between interfacial layers and the bulk electrolyte. Equilibration and production times should consequently be assessed against the slowest structural and dynamical processes relevant to the observable being calculated, and statistical uncertainties should be reported whenever possible.

Force-field parametrization introduces another, independent level of model dependence. Nonpolarizable force fields enable long simulations and systematic comparisons but represent electronic polarization only implicitly through their parametrization, whereas explicitly polarizable models increase physical fidelity at additional computational cost. The resulting ion–ion and ion–electrode interactions determine adsorption, molecular orientation, transport, and electric double-layer structure and can therefore affect the calculated capacitance even when the same electrode-charging method is employed. Conversely, increasing the sophistication of the electrode model does not eliminate inaccuracies originating from the electrolyte force field. Quantitative agreement with experiment thus requires balanced descriptions of both sides of the interface rather than refinement of the electrode response alone.

These limitations are strongly coupled. Increasing the simulation cell reduces finite-size artifacts but increases computational cost and may restrict the accessible trajectory length; using polarizable or electronic-structure-aware models improves the physical description but may further reduce the system sizes and time scales that can be sampled. Likewise, longer trajectories cannot compensate for an inappropriate force field or electrostatic boundary condition. Reliable simulations, therefore, require convergence to be considered along several independent axes—system dimensions, electrode thickness, electrostatic treatment, equilibration and sampling time, and interaction model—rather than through a single criterion. This multidimensional dependence is one reason why quantitative discrepancies between independent studies cannot generally be attributed to one methodological choice alone.

## 3 Results of Graphene/Ionic-Liquid Supercapacitor Simulations

This section reviews the main results reported in the literature for graphene and graphene-derived supercapacitors with ionic-liquid electrolytes, covering interfacial structure; electrode engineering through curvature, defects, and doping; nanoconfinement and anomalous capacitance; and the emerging use of machine-learning interatomic potentials. Table 2 summarizes the electrode, electrolyte, method, and reported capacitance for the main studies discussed.

**Table 2:** Summary of Selected Studies Discussed in This Section

**Electrode**	**Electrolyte**	**Method**	**Capacitance/finding**	**ref.**
Graphene	neat or 10 mol % [BMIM][BF_4_]/[BMIM][TFSI] + cosolvent	CPM	camel-shaped *C*–*V* profile retained after dilution	Tu and McDaniel^25^
Graphite slit electrodes	[BMIM][NTF_2_]	CPM	4.0–4.4 μF/cm^2^; EDL disrupted under strong confinement	Bonnaud and Shiba^73^
Graphene	[C_2_MIM][NTF_2_], [N_4,1,1,1_][NTF_2_]	CPM	similar capacitance despite different structure and dynamics	Demir and Searles^74^
Graphene, graphyne	[BMIM][PF_6_], [EMIM][BF_4_],[CH][GLY], [PYR][TFSI]	FCM	graphyne generally increases capacitance by 3–14%	Chagas et al.^35^
Graphene	hydrated [BMIM] + ${\mathrm{NO}}_{3}^{-}$ / ${\mathrm{ClO}}_{4}^{-}$ /Br^–^	FCM	2.30–2.55 μF/cm^2^; 5.03–5.58 J/g	Silva and Colherinhas^75^
Graphene	[EMIM][BF_4_] + water/methanol	FCM	similar capacitance; methanol raises energy density by ∼38%	Silva et al.^76^
Graphene	hydrated [BMIM][Cl]/[BMIM][Br] mixtures	FCM	minor charge changes; ∼9% higher energy density with chloride	Oliveira et al.^77^
Graphene	hydrated amino-acid–based ILs	FCM	2.04–2.71 μF/cm^2^; competitive biodegradable electrolytes	Silva et al.^78^
Graphene, graphyne	[EMIM][BF_4_], NaCl(aq)	FCM	graphyne: higher capacitance and gravimetric energy density	Chagas et al.^79^
Few-layer graphene	ionic liquid	Exp. (FET)	compact, ion-dense EDL	Uesugi et al.^21^
HOPG	ionic liquid	Exp. (AFM)	bias-dependent EDL reconfiguration	Black et al.^22^
Graphite	[EMIM][TFSI]	Exp. (AFM)	resolved Stern-layer unit cell	Elbourne et al.^23^
Graphene	ionic liquid	Exp. (force)	subnanometer EDL structure	Jurado et al.^24^
Onion-like carbon	ionic liquid	FCM	capacitance increases with curvature	Feng et al.^27^
Carbon nanotube	[BMIM][PF_6_]	MD + DFT	curvature increases EDL and quantum capacitance	Paek et al.^97^
N-doped graphene	[BMIM][PF_6_]	MD + DFT	doping increases quantum, but not EDL, capacitance	Paek et al.^28^
C_60_-decorated graphene	ionic liquid	FCM	up to 150% capacitance enhancement; asymmetric EDL response	Fileti and Colherinhas^100^
Metallic slit pore	model ionic liquid	Theory^a^	image screening enables a superionic state	Kondrat and Kornyshev^19^
Graphene slit pore	confined ions	DFT^a^	quantum capacitance depends on pore width, ion, and doping	Gameel et al.^112^
Imidazolium/pyrrolidinium ILs	bulk ILs and mixtures	MLIP	transferable to compositions excluded from training	Goodwin et al.^117^
Graphite	[PYR_13_][TFSI]	CPM	asymmetric *C*–*V* response; capacitance decreases with temperature	Vatamanu et al.^80^
Graphene oxide	[EMIM][BF_4_]	FCM	capacitance decreases with oxidation	DeYoung et al.^103^
Carbide-derived carbon	organic electrolyte	Exp.	anomalous capacitance increase below 1 nm	Chmiola et al.^109^
Slit nanopore	ionic liquid	Atomistic MD	U-shaped capacitance scaling for 0.75–1.26 nm pores	Wu et al.^110^
Graphene + integrated conductor	ionic liquid	FCM	additional EDLs modify potential distribution and energy storage	Chagas et al.^107^

a Included as a theoretical or first-principles complement to the classical molecular dynamics literature discussed in this review.

The studies discussed in this section were selected to illustrate representative combinations of simulation methodology, electrolyte chemistry, and electrode structure rather than to provide an exhaustive catalog of every graphene–electrolyte system reported in the literature. Particular emphasis is placed on studies that isolate physically informative variables, such as ion identity, electrode curvature, defects, doping, or confinement; or that demonstrate how the methodological choices introduced in Section 2 affect the interpretation of interfacial structure, charging, and capacitance. The examples should therefore be read as representative case studies connecting molecular-scale mechanisms to modeling strategy, while Table 2 provides a compact comparison of the principal systems considered.

### 3.1 Structure of the Graphene/Ionic-Liquid Interface

Before turning to specific results, it is useful to survey the chemical space of ionic liquids that the studies discussed throughout this review actually explore. Figure 4 collects the cation and anion families involved, spanning imidazolium, pyrrolidinium, quaternary ammonium, and choline cations, paired with a wide range of anions from small inorganic species to larger fluorinated and amino-acid-based ones. This diversity is a recurring theme of Section 3: as shown below, anion identity in particular is often the single variable that most strongly controls electric double-layer structure and capacitance for an otherwise fixed graphene electrode.

**Figure 4:**
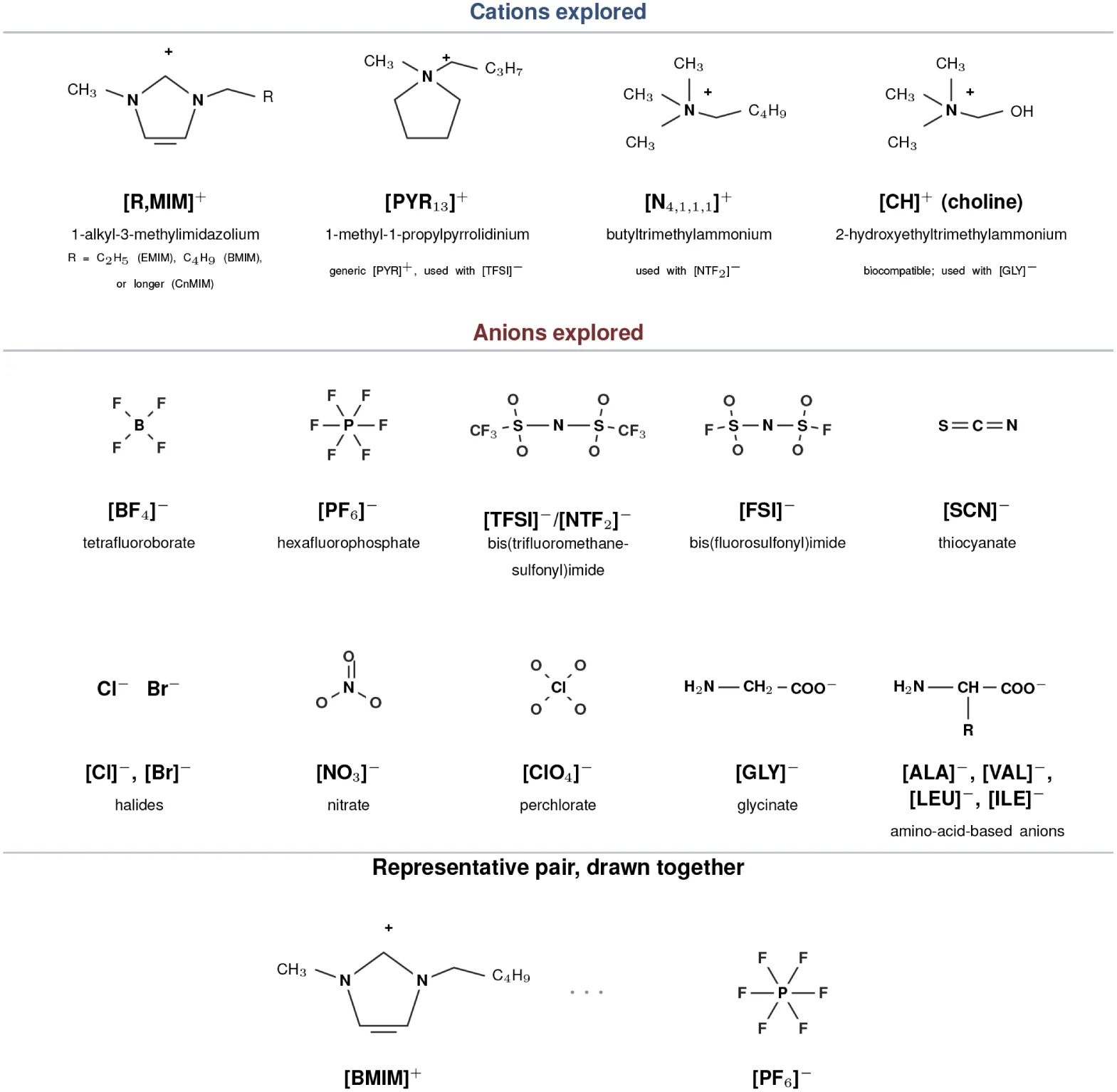
Cation and anion families explored in this review. Skeletal structures are schematic, drawn to illustrate connectivity rather than exact bond geometry. Imidazolium cations (1-alkyl-3-methylimidazolium) cover [EMIM]^+^, [BMIM]^+^, and the general [CnMIM]^+^ family; pyrrolidinium,quaternary ammonium, and choline cations appear in specific comparative studies. Anions range from small fluorinated species ([BF_4_]^−^, [PF_6_]^−^, [TFSI]^−^/[NTF_2_]^−^, [FSI]^−^, [SCN]^−^) to halides, oxoanions, and the biodegradable glycinate and amino acid–based anions used in the studies discussed below. The bottom panel shows one representative pair, [BMIM]­[PF_6_], drawn together as a reminder that capacitance and energy density depend on the specific cation–anion combination, not on either ion in isolation.

The overscreening-to-crowding crossover introduced in Section 1 has a direct microscopic signature at the graphene surface. The local ion density oscillates with distance from the electrode, alternating between layers dominated by counterions and layers dominated by co-ions, before decaying to the bulk value within one to two nanometers, as highlighted in Figure 5. This section examines how this layered structure has been characterized, both computationally and experimentally, at graphene and graphite electrodes, specifically.

**Figure 5:**
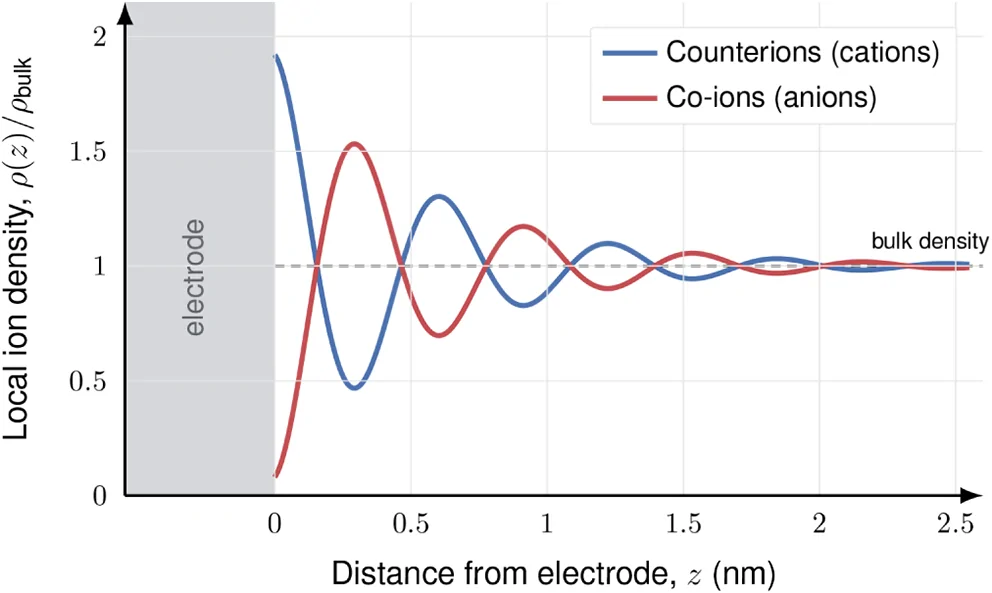
Schematic local ion density as a function of distance from the electrode surface, *z*, normalized to the bulk value. Counterions (blue) accumulate in a first layer immediately adjacent to the electrode, followed by a depleted, co-ion-rich (red) second layer, with the oscillation decaying to the bulk value within one to two nanometers. This alternating, overscreened structure is the direct microscopic signature of the overscreening-to-crowding crossover discussed in Section 1, and is the structural motif that the studies summarized in this section resolve computationally and probe experimentally at graphene and graphite electrodes. Curves are illustrative, not extracted from a specific simulation.

Tu and McDaniel^25^ used constant-voltage molecular dynamics to investigate the differential capacitance of [BMIM]­[BF_4_] and [BMIM]­[TFSI] at model graphene electrodes, both as neat ionic liquids and as solutions containing 10 mol % ionic liquid in acetonitrile or 1,2-dichloroethane. Notably, dilution did not restore the bell-shaped capacitance profile conventionally associated with the classical dilute-solution limit. Instead, both the neat and the 10 mol % electrolytes exhibited camel-shaped profiles, characterized by a minimum at the potential of zero charge and two flanking maxima, as schematically illustrated in Figure 6. The authors attributed this behavior to electric-field-induced reorganization within the innermost interfacial layer. Near the potential of zero charge, nonpolar butyl and trifluoromethyl groups preferentially occupy the region closest to graphene. Increasing the magnitude of the electrode potential favors the approach of polar and charged moieties, increasing the inner-layer charge density and, consequently, the differential capacitance. These results demonstrate that electrolyte concentration alone does not determine the qualitative shape of the capacitance–potential curve: even a solvent-rich electrolyte can retain ionic-liquid-like behavior when its interfacial layer remains governed by specific molecular orientations and ion–electrode interactions.

**Figure 6:**
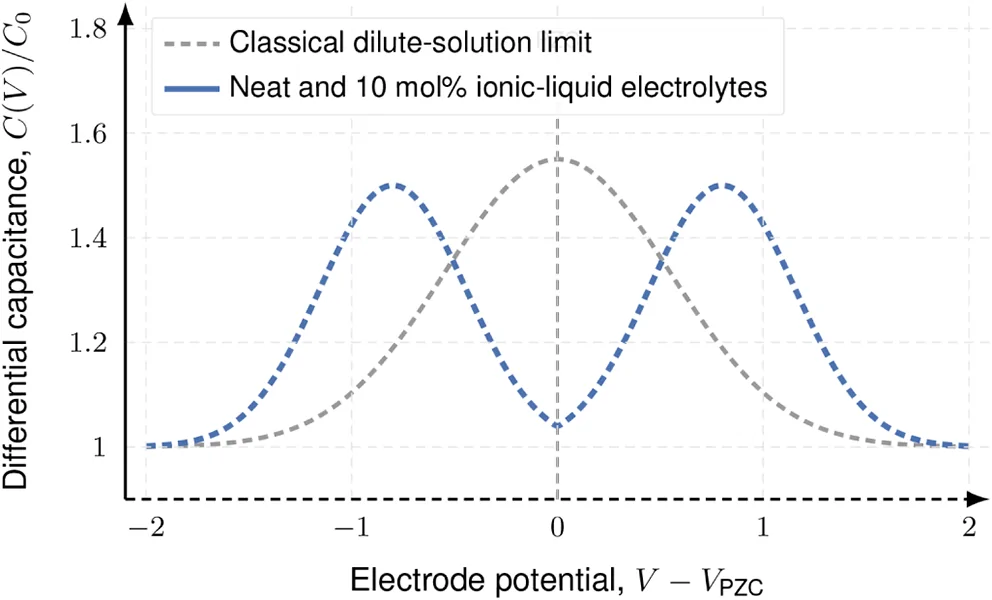
Schematic differential capacitance as a function of the electrode potential relative to the potential of zero charge (PZC). The gray dashed curve represents the idealized bell-shaped response associated with the classical dilute-solution limit. In contrast, both the neat and 10 mol % ionic liquid electrolytes investigated by Tu and McDaniel^25^ exhibited camel-shaped profiles, qualitatively represented by the blue curve, with a minimum at the PZC and two maxima at finite electrode polarization. The curves are illustrative and are not extracted from a specific simulation.

Bonnaud and Shiba^73^ used constant-potential molecular dynamics to investigate the electric double layer formed by [BMIM]­[NTF_2_] confined between planar graphite electrodes over a wide range of electrode separations, applied potentials, and temperatures. The authors found average differential capacitances of approximately 4.0–4.4 μF/cm^2^, in agreement with previous molecular simulations, and showed that the capacitance depends systematically on both confinement and temperature. Under strong nanoconfinement, overlapping electric double layers disrupt the interfacial structure and reduce the capacitance, whereas larger electrode separations allow fully developed double layers extending roughly 3–4 nm from the electrode surface, with a bulk-like region forming between them. Increasing the applied potential enhances ionic segregation and interfacial layering, while higher temperatures facilitate this reorganization and generally increase the differential capacitance. The authors further related the dependence of the fluid contribution to the differential capacitance on electrode separation to a Langmuir-like sorption behavior, highlighting the intimate connection between electric-double-layer formation and ion adsorption.

A complementary perspective was provided by Elliott et al.^44^ using QMMD simulations of aqueous LiCl, NaCl, KCl, MgCl_2_, and CaCl_2_ electrolytes at dynamically polarizable graphene electrodes. Although the interfacial adsorption mechanisms varied substantially across the cation series, including partial dehydration and direct inner Helmholtz layer adsorption of K^+^, the computed electric double-layer capacitance remained comparatively insensitive to cation identity. This result illustrates that pronounced changes in microscopic interfacial organization do not necessarily translate into equally pronounced changes in macroscopic capacitance, while also highlighting the importance of allowing the graphene charge distribution to respond dynamically to local ion adsorption.

Demir and Searles^74^ used constant-potential molecular dynamics to compare the graphene/electrolyte interface formed by two ionic liquids, [C_2_MIM]­[NTF_2_] and [N_4,1,1,1_]­[NTF_2_], whose markedly different viscosities lead to distinct interfacial dynamics. The authors found that changing the cation substantially altered the electric double-layer structure and the preferred orientation of ions at the graphene surface, with both cations tending to align approximately parallel to the negatively charged electrode. The higher-viscosity [N_4,1,1,1_]­[NTF_2_] exhibited ionic mobilities nearly 1 order of magnitude lower than those of [C_2_MIM]­[NTF_2_], leading to correspondingly slower charging dynamics. Despite these pronounced structural and dynamic differences, the overall electrode charge distribution and the resulting capacitance remained remarkably similar for the two electrolytes. Charging proceeded primarily through the rearrangement and reorientation of ions already located within approximately 1 nm of the electrode, rather than by long-range diffusion from the bulk, reaching steady-state charge profiles on time scales of about 10 ns that increased with the viscosity of the ionic liquid.

The electrode topology provides a separate means of modifying interfacial capacitance. Chagas et al.^35^ employed the fixed-charge method (FCM) to compare graphene and graphyne electrodes paired with four ionic liquids, [BMIM]­[PF_6_], [EMIM]­[BF_4_], [CH]­[GLY], and [PYR]­[TFSI], over an electrochemical window of approximately 2.0 V. Across nearly all electrolytes, graphyne exhibited a higher double-layer capacitance than graphene, with improvements ranging from 3 to 14%; the only exception was [PYR]­[TFSI], for which a slight reduction of about 1% was observed. The largest performance gains were obtained for [EMIM]­[BF_4_] and the biocompatible [CH]­[GLY], the latter retaining capacitances comparable to those of [EMIM]­[BF_4_] while offering a more sustainable electrolyte alternative. The simulations further showed that replacing graphene with graphyne has only a minor influence on ion mobility, indicating that the enhanced capacitance originates primarily from changes in the electrode/electrolyte interface rather than from altered ionic transport. An exploratory analysis also suggested that hybrid graphene/graphyne supercapacitors, with graphyne as the negative electrode and graphene as the positive electrode, may further improve the double-layer capacitance, highlighting a promising direction for future computational and experimental studies.

The same graphene electrode has also been examined with more realistic hydrated ionic-liquid compositions. Silva and Colherinhas^75^ employed the fixed-charge method to investigate [BMIM]-based ionic liquids containing nitrate, perchlorate, and bromide anions, both as pure electrolytes and in binary mixtures, as illustrated in Figure 7. The results showed that anion identity plays a decisive role in determining the electric-double-layer structure and, consequently, the electrochemical performance of the supercapacitor. Among the pure electrolytes, [BMIM]­[Br] exhibited the highest capacitance (2.55 μF/cm^2^) and projected gravimetric energy density (5.58 J/g), despite displaying the weakest overall electrode–electrolyte interaction. This behavior was attributed to the small size and high mobility of Br^–^, which favor the formation of a compact double layer. By contrast, ${\mathrm{NO}}_{3}^{-}$ produced intermediate performance by stabilizing the interfacial structure through hydrogen bonding with water, whereas ${\mathrm{ClO}}_{4}^{-}$ yielded the lowest capacitance and energy density despite interacting more strongly with the electrode. The binary mixtures further demonstrated that combining complementary anion properties can improve device performance. In particular, the ${\mathrm{Br}}^{-}/{\mathrm{NO}}_{3}^{-}$ mixture combined the high mobility of bromide with the interfacial structuring ability of nitrate, whereas the other mixtures exhibited little or no synergistic behavior. These results identify anion composition as an important design parameter for hydrated ionic-liquid supercapacitors.

**Figure 7:**
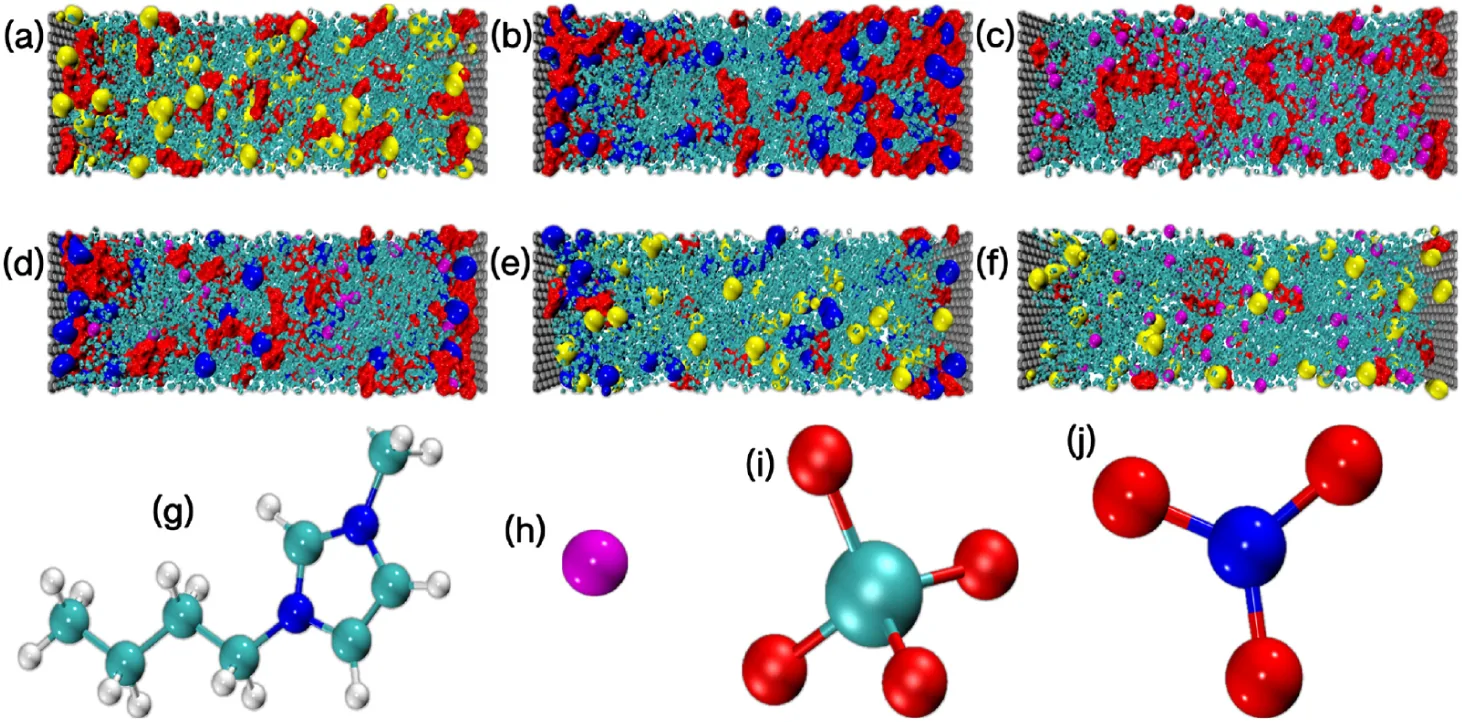
Representative molecular dynamics models employed for graphene-based supercapacitors containing pure and mixed hydrated ionic liquids. Panels (a–f) show the six electrolyte configurations investigated. The molecular structures of the electrolyte components are shown in (g) the *N*-butyl-*N*-methylimidazolium ([BMIM]^+^) cation; (h) Br^–^; (i) ${\mathrm{ClO}}_{4}^{-}$ ; and (j) ${\mathrm{NO}}_{3}^{-}$ . In the molecular dynamics models, [BMIM]^+^ is shown in red, ${\mathrm{NO}}_{3}^{-}$ in yellow, ${\mathrm{ClO}}_{4}^{-}$ in blue, Br^–^ in pink, and water molecules in cyan. Reproduced from Silva and Colherinhas^75^ under the terms of the CC BY 4.0 license.

The influence of cosolvents on graphene/ionic liquid interfaces has also been examined directly. As discussed above, the constant-potential simulations of Tu and McDaniel^25^ showed that the camel-shaped differential capacitance profile of [BMIM]­[BF_4_] and [BMIM]­[TFSI] at graphene is retained even at an ionic liquid mole fraction of 0.10, so that substantial dilution does not necessarily suppress the underlying potential-dependent charging response. De Silva et al.^76^ subsequently investigated [EMIM]­[BF_4_] electrolytes cosolvated with water and methanol using the fixed-charge method. Both cosolvents substantially reorganized the electric double layer by modifying the hydrogen-bonding network and competing with the ions for adsorption sites at the graphene surface. Methanol exhibited particularly strong preferential adsorption at both positively and negatively charged electrodes, partially displacing ions and water from the interfacial region and altering the corresponding charge density profiles. Although the total capacitance remained nearly unchanged across the investigated compositions, the reduced electrolyte mass and modified interfacial organization improved the projected device-level energy density. At an operating potential of 2.5 V, the methanol-rich electrolyte produced a gravimetric energy density approximately 38% higher than that of the neat ionic liquid while reducing the total device mass by ∼22%. Thus, the principal benefit of methanol addition was not an increase in capacitance but an improvement in gravimetric energy-storage performance through solvent-induced restructuring and mass reduction.

Together, these studies show that dilution should not be viewed simply as a monotonic reduction of capacitive performance with decreasing ionic liquid content. Cosolvents can substantially modify interfacial organization, transport properties, and electrolyte mass, while preserving important features of the capacitive response. Direct quantitative comparison nevertheless remains difficult because the studies employ different ionic liquids, cosolvents, and electrode charging protocols.

Oliveira et al.^77^ examined a related composition effect by varying the relative amounts of chloride and bromide in hydrated [BMIM]-based electrolytes at graphene electrodes. Changing the anion composition produced only minor differences in the electrostatic charge distribution at the electrode, indicating that the overall electric double-layer response remained comparatively stable. The effect was more pronounced at the device level, however, increasing the chloride concentration increased the projected gravimetric energy density by approximately 9% without compromising the electrochemical stability of the system. This result shows that anion substitution can improve a mass-normalized performance metric even when its influence on interfacial charge storage is limited, which is a distinction that is particularly relevant when evaluating electrolytes for weight-sensitive applications.

Taken together, these composition studies show that modifying the electrolyte does not affect all performance metrics through the same microscopic mechanisms. Changes in anion identity can directly reorganize the electric double layer and alter capacitance when they modify ion size, mobility, hydrogen-bonding interactions, or interfacial packing, as observed for the hydrated [BMIM]-based electrolytes. By contrast, changing the solvent or the relative abundance of chemically related anions may leave the interfacial charge-storage response comparatively unchanged while still improving gravimetric energy density through reductions in electrolyte mass or changes in accessible operating conditions. Consequently, electrolyte modifications that improve device-level energy density should not automatically be interpreted as improvements in the intrinsic interfacial capacitance. This distinction also cautions against ranking electrolytes solely from a single performance metric: composition, interfacial organization, capacitance, and gravimetric energy density are related but not interchangeable descriptors of electrochemical performance.

De Silva et al.^78^ investigated amino acid-based ionic liquids (AAILs) as biodegradable electrolytes for graphene-based supercapacitors, using the fixed-charge method. Employing [EMIM]-based ionic liquids with alanine, valine, leucine, and isoleucine anions, the authors demonstrated that electrolyte hydration plays a key role in determining the electric double-layer structure and device performance, leading to a nonlinear dependence of the potential difference on water content. Depending on the electrolyte composition, the calculated capacitances ranged from 2.04 to 2.71 μF/cm^2^, while gravimetric energy-density calculations indicated that these biodegradable electrolytes remain competitive with conventional ionic liquids. Beyond introducing a new family of sustainable electrolytes, the study provides one of the first systematic assessments of how hydration influences the electrochemical behavior of amino acid-based ionic liquids, highlighting electrolyte hydration as an additional design parameter for environmentally friendly supercapacitors. Chagas et al.^79^ later extended the graphene/graphyne comparison discussed above^35^ to symmetric and asymmetric electrode configurations containing either [EMIM]­[BF_4_] or aqueous NaCl electrolytes. The graphyne-based devices generally exhibited higher capacitance than their graphene counterparts, reaching approximately 2.4 μF/cm^2^ at 2 V. Because graphyne also has a lower electrode mass, this capacitance enhancement translated into a higher gravimetric energy density.

Temperature is a further variable that governs the graphene-ionic liquid interface. Vatamanu et al.^80^ used molecular dynamics simulations to investigate the differential capacitance of the ionic liquid [PYR_13_]­[TFSI] at graphite electrodes over a range of electrode potentials and temperatures. The simulations revealed that the multilayer electric double-layer structure extends several nanometers from the electrode and that both ion orientation and interfacial composition evolve systematically with the applied potential. Near the potential of zero charge, cations and anions in the first adsorbed layer preferentially orient perpendicular to the surface, whereas increasing polarization drives the counterions to adopt progressively more parallel configurations. The resulting differential capacitance curve exhibits pronounced asymmetry with respect to electrode polarity, arising from the distinct adsorption characteristics and molecular geometries of the cation and anion, and the capacitance maxima correspond to the onset of interfacial ion crowding. Increasing temperature decreases the differential capacitance and suppresses the minimum near the potential of zero charge, reflecting the growing importance of entropic and excluded-volume effects in the crowded interfacial layer. The same authors extended this approach to a pyrrolidinium-based ionic liquid with a bis­(fluorosulfonyl)­imide anion at graphite electrodes^81^ and, separately, examined how surface topography modulates these temperature effects.^82^

For a different ionic liquid, Liu et al.^83^ investigated the temperature-dependent differential capacitance of [BMIM]­[PF_6_] at graphite electrodes and found asymmetric camel-shaped capacitance curves with systematically higher capacitance under negative polarization, reflecting the specific adsorption of [BMIM]^+^ cations. As the temperature increases, the negative-potential capacitance maximum decreases monotonically owing to progressive thickening of the electric double layer, whereas the positive-potential maximum first increases and then decreases as a result of the competition between weakened specific adsorption and improved electrostatic screening. Extending the discussion beyond uniform heating, Prakash and Sathian^84^ demonstrated that thermal gradients can qualitatively modify the charging response of graphene-based supercapacitors. For [BMIM]­[BF_4_], increasing the thermal gradient transforms the differential capacitance profile from a U shape to a bell shape, whereas [BMIM]­[PF_6_] retains a U-shaped response with an overall increase in capacitance. The authors related these distinct trends to gradient-induced changes in the interfacial ion organization and charge screening.

More recently, Chagas and Colherinhas^85^ examined graphene/[EMIM]­[BF_4_] supercapacitors over the temperature range of 300–600 K using the fixed-charge method and found that, despite the increase in ionic mobility with temperature, the electric double layer remains remarkably stable. As a consequence, capacitance varied by less than about 13%, while the projected gravimetric energy density increased by approximately the same amount at high temperature, supporting the use of graphene-based supercapacitors in thermally demanding applications.

The different temperature dependences reported in these studies therefore indicate that there is no universal thermal response of the capacitance at graphene and graphite/ionic liquid interfaces. Increasing temperature simultaneously affects ionic mobility, adsorption, molecular orientation, interfacial packing, and electrostatic screening, and the balance among these contributions depends on the particular cation–anion pair and electrode environment. Thus, the decrease in capacitance reported for [PYR_13_]­[TFSI], the polarity-dependent and nonmonotonic response obtained for [BMIM]­[PF_6_], and the comparatively weak variation observed for [EMIM]­[BF_4_] should not necessarily be regarded as contradictory results. Rather, they demonstrate that temperature acts through electrolyte-specific changes in electric double layer organization. Quantitative comparison is further complicated by differences in electrode models and simulation protocols among these studies, so the most transferable conclusion is that thermal effects must be assessed for the specific electrode–electrolyte combination rather than treated as a universal correction to capacitance.

The chemical identity of the ions themselves also shapes the interface. Cation structure provides a particularly clear example of the nontrivial relationship between electrode–ion affinity and capacitance. Molecular dynamics simulations of imidazolium-based ionic liquids with different alkyl-chain lengths showed that increasing the chain length strengthens interactions with the graphene surface while reducing the differential capacitance.^86^ This result demonstrates that stronger electrode–ion interactions do not necessarily translate into more efficient charge storage, because molecular orientation, steric occupation, and packing within the first interfacial layers also contribute to the electrochemical response. Jo et al.^87^ employed molecular dynamics simulations to investigate graphene supercapacitors containing [EMIM]-based ionic liquids with four cyano-functionalized anions: [SCN]^−^, [N­(CN)_2_]^−^, [C­(CN)_3_]^−^, and [B­(CN)_4_]^−^. All systems developed the characteristic layered electric-double-layer structure and exhibited asymmetric camel-shaped differential-capacitance curves with maxima at negative electrode potentials, as shown in Figure 8. While the peak capacitances remained comparable across the four electrolytes, the corresponding peak shifted systematically toward more negative potentials as the number of cyano groups on the anion increased. By resolving the contributions of successive ionic layers, the authors demonstrated that the differential-capacitance behavior is governed primarily by ion exchange between the electric double layer and the bulk electrolyte, with the charging mechanism depending strongly on the applied potential. In particular, the capacitance maxima originate from the rapid desorption of anions from the interfacial layer, except for [EMIM]­[SCN], where both cation and anion exchange contribute significantly to the charging process.

**Figure 8:**
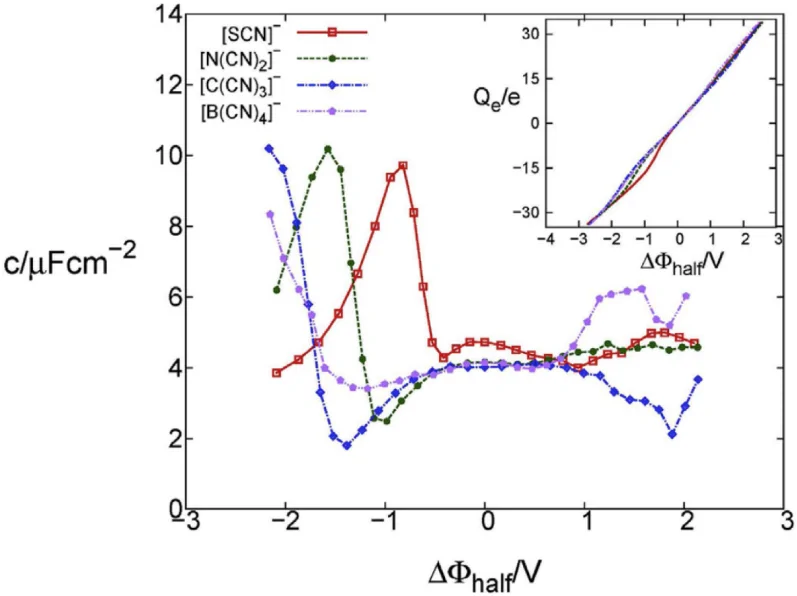
Differential capacitance as a function of the half-cell potential for graphene electrodes in four cyano-containing ionic liquids. Although the maximum differential capacitance remains similar among the electrolytes, increasing the number of cyano groups shifts the capacitance maximum toward more negative potentials, demonstrating that the electrolyte anion strongly influences the charging mechanism at the graphene/electrolyte interface. Reproduced with permission from Jo et al.,^87^ *Electrochimica Acta* **284** (2018) 577–586. Copyright © 2018 Elsevier B.V.

The importance of electrode morphology has also been demonstrated independently for [EMIM]­[BF_4_] confined by graphene-based electrodes. Molecular dynamics simulations of vertically oriented graphene showed that changes in surface morphology modify the distribution and migration of interfacial ions and consequently affect the differential capacitance response.^88^ These observations demonstrate that even when the electrolyte composition is held fixed, atomic-scale changes in electrode geometry can substantially reorganize the electric double layer.

The relative importance of ion identity and electrode morphology was examined more directly by Hu et al.,^89^ who used molecular dynamics simulations to investigate the electric double-layer structure and differential capacitance of the ionic liquids [BMIM]­[BF_4_] and [BMIM]­[PF_6_] at graphite and gold electrodes with distinct surface morphologies. For a given electrode, both electrolytes produced remarkably similar interfacial structures and differential capacitance curves, indicating that substituting [BF_4_]^−^ with [PF_6_]^−^ has only a minor influence on the electrochemical response. In contrast, the atomic structure of the electrode surface proved to be a dominant factor: atomically flat surfaces, such as basal graphite and Au(001), exhibited nearly constant capacitances of approximately 5 μF/cm^2^ over the electrochemical stability window, whereas corrugated surfaces, including prismatic graphite and Au(011), displayed a much stronger potential dependence. The authors attributed this behavior to the interplay between surface roughness and ion size, highlighting electrode morphology as a key determinant of electric double-layer capacitance.

Comparison with the composition-dependent studies discussed above shows, however, that the relative importance of electrolyte chemistry and electrode morphology is not universal. Substitution among anions can strongly affect the interfacial response when it substantially changes ion size, mobility, adsorption affinity, hydrogen-bonding interactions, or molecular geometry. In contrast, Hu et al.^89^ found only minor differences between [BF_4_]^−^ and [PF_6_]^−^ for a given electrode, while changing the atomic morphology of the surface produced a much stronger variation in differential capacitance. These observations are not necessarily inconsistent: they indicate that the dominant control parameter depends on how strongly a given modification perturbs the first adsorbed layers. In some systems this perturbation originates primarily from electrolyte chemistry, whereas in others the geometric constraints imposed by the electrode surface dominate ion packing and charge screening. This system dependence helps explain why electrolyte rankings obtained for one electrode morphology cannot be assumed to remain unchanged for another.

Wang and Tian^90^ extended the analysis of anion-dependent interfacial organization to a metallic electrode by comparing [BMIM]­[BF_4_], [BMIM]­[PF_6_], and [BMIM]­[TFSI] at neutral and charged Au(100) surfaces using atomistic molecular dynamics. All three ionic liquids formed pronounced interfacial layers, but the position, composition, and molecular orientation of the innermost layer depended strongly on the geometry and electronic structure of the anion. The relatively compact and symmetric ${\mathrm{BF}}_{4}^{-}$ and ${\mathrm{PF}}_{6}^{-}$ anions exhibited interfacial arrangements distinct from those of the larger, flexible, and charge-delocalized TFSI^–^ anion, particularly in their orientational response to surface charging. Although the cation was identical in all systems, its interfacial distribution was also modified indirectly through anion–cation correlations and competition for adsorption near the gold surface. These results show that anion selection controls not only which species occupies the first adsorbed layer but also how the entire interfacial ionic network responds to electrode polarization. The qualitative importance of anion geometry therefore extends beyond carbon electrodes, although the specific adsorption structures observed at Au(100) should not be transferred quantitatively to graphene because of the different ion–surface interactions.

Fang and Smolyanitsky^91^ employed all-atom molecular dynamics simulations to investigate mixtures of [EMIM]­[TFSI] and [EMIM]­[BF_4_] at graphene electrodes. As expected, the integral capacitance of the mixtures generally assumed values intermediate between those of the two pure ionic liquids. Nevertheless, analysis of the interfacial ion populations revealed a more complex charging mechanism than a simple interpolation between the end members. Near uncharged electrodes, the larger [TFSI]^−^ anion adsorbs preferentially owing to its stronger van der Waals interaction with graphene, whereas electrode charging induces an effective exchange between [TFSI]^−^ and [BF_4_]^−^ within the first adsorbed layer.

This anion-exchange mechanism enhances cation adsorption and increases the capacitance of the negative electrode, highlighting how differences in ion size and ion–electrode interactions can be exploited to tailor the charging response of mixed ionic-liquid electrolytes. A related molecular-dynamics study by Li et al.^92^ investigated binary ionic-liquid mixtures at onion-like carbon electrodes and showed that the differential capacitance is largely independent of the applied potential but increases with temperature, reflecting the dominant role of electric-double-layer thickness in determining the thermal response of these mixed-electrolyte systems.

Noh and Jung^93^ combined equilibrium and nonequilibrium molecular dynamics simulations to investigate the molecular mechanisms governing the charging dynamics of graphene-based electric double-layer capacitors containing [EMIM]­[SCN], as illustrated in Figure 9. Their equilibrium simulations revealed a pronounced layered organization of the ionic liquid near the graphene surface, with abrupt, potential-induced rearrangements of these interfacial layers, giving rise to regions of enhanced differential capacitance. The nonequilibrium simulations further showed that the charging response is controlled by the collective reorganization of the interfacial electrolyte rather than by the simple accumulation of counterions at the electrode. In particular, the evolution of the electrode charge was strongly correlated with ion displacement perpendicular to the graphene surface, and charging occurred predominantly through the desorption of co-ions rather than through the adsorption of counterions. Importantly, ions initially located in the bulk electrolyte contributed substantially to the stored charge, challenging the conventional picture in which charging is governed primarily by ions already present in the first interfacial layer. These results demonstrate that the charging rate and capacitance are closely coupled to cooperative ion transport and restructuring across multiple ionic layers. Nevertheless, because these mechanisms were identified for a specific ionic liquid and an idealized graphene interface, their quantitative relevance to other electrolytes and structurally heterogeneous electrodes remains to be established.

**Figure 9:**
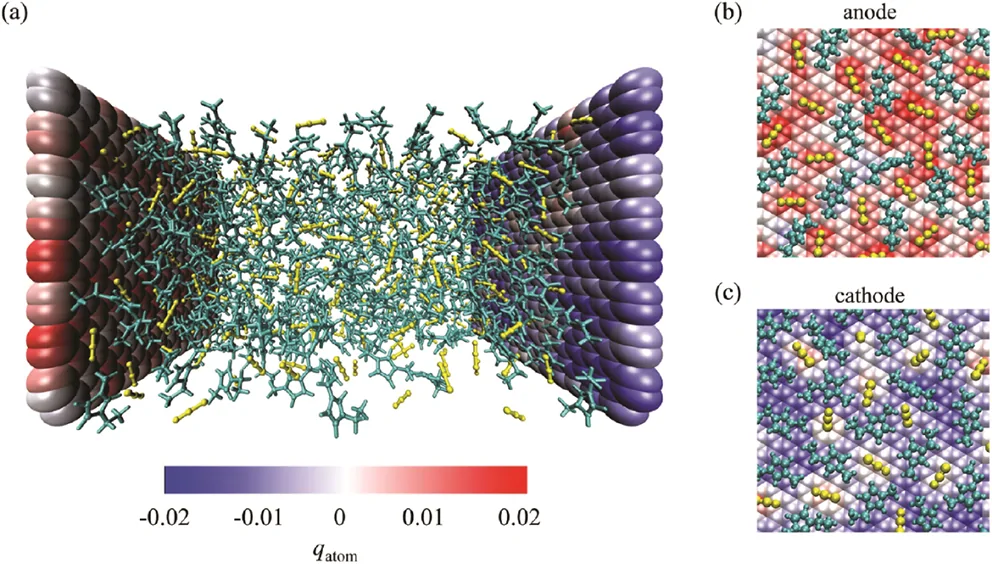
Representative simulation snapshot of the graphene/[EMIM]­[SCN] electric double-layer capacitor at a potential difference ΔΨ = 2 V. The charge of each electrode atom is indicated by color (scale bar in units of *e*); the left electrode is the anode and the right is the cathode. [EMIM]^+^ and [SCN]^−^ ions, shown in cyan and yellow, fill the 6 nm gap between the electrodes as the electrolyte. Reproduced from ref.^93^ with permission from the Royal Society of Chemistry.

The charging mechanism inferred by Noh and Jung^93^ also provides an instructive contrast with the behavior reported by Demir and Searles.^74^ In the latter study, charging was dominated by rearrangement and reorientation of ions already located within approximately 1 nm of the graphene surface, whereas Noh and Jung^93^ found that ions initially located in the bulk contributed substantially to the stored charge and that co-ion desorption played a central role. This difference indicates that the spatial origin of the ions participating in charging is itself electrolyte-dependent and should not be reduced to a universal adsorption mechanism. Ion viscosity, molecular structure, and the initial organization of the interfacial layers are therefore likely to influence whether charging proceeds predominantly through local rearrangement or longer-range ion exchange. Because the available studies simultaneously differ in electrolyte composition and simulation protocols, however, the contribution of each factor cannot yet be isolated quantitatively. Controlled comparisons in which the electrolyte or the charging protocol is varied independently would be required to establish a general mechanistic criterion.

One of the earliest systematic molecular dynamics studies of graphene-based supercapacitors was reported by Shim et al.,^94^ who compared neat [EMIM]­[BF_4_] with its acetonitrile solution as electrolytes. The authors showed that pure ionic liquids provide specific capacitances approximately 55–60% higher than those of the organic electrolyte because of more efficient screening of the electrode charge. They also identified a pronounced cathode–anode asymmetry arising from differences in ion size and molecular geometry, while demonstrating that the electrochemical performance of the ionic liquid remains remarkably stable over a broad temperature range. These results established graphene as an important model electrode for molecular simulations of ionic-liquid supercapacitors.

Merlet et al.^95^ validated a coarse-grained representation of [BMIM]­[PF_6_] for simulations of ionic-liquid/graphite interfaces. Besides accurately reproducing the experimental surface tension, the model yielded differential capacitances and interfacial structures in excellent agreement with all-atom simulations, demonstrating that coarse-grained force fields can preserve the essential physics of electric double-layer formation while enabling simulations of larger and more complex electrode architectures. Finally, Chagas et al.^96^ investigated aqueous mixtures of the biodegradable ionic liquid [CH]­[GLY] with [BMIM]­[PF_6_] at graphyne electrodes by using atomistic molecular dynamics. Although increasing the concentration of [BMIM]­[PF_6_] reduced the total capacitance, the cholinium-based electrolytes remained competitive with conventional ionic liquids while offering substantially lower toxicity. The study also showed that graphyne consistently outperforms graphene, reinforcing its potential as an alternative electrode material for sustainable supercapacitors.

Taken together, these studies establish a consistent qualitative picture in which ion identity, molecular orientation, solvent content, temperature, and electrode morphology jointly determine the structure and dynamics of the graphene/ionic liquid interface. Quantitative comparisons across studies should nevertheless be made with caution, because the reported capacitances were obtained using different electrode-charging methods, force fields, potential windows, electrolyte compositions, and definitions of device-level performance. Fixed-charge simulations provide valuable comparative trends but neglect the instantaneous polarization of the electrode, whereas constant-potential studies commonly retain ideal-metal and nonpolarizable electrolyte approximations. Consequently, the most robust conclusions are generally the relative trends obtained within a common simulation framework, rather than absolute rankings assembled from values reported by different studies. Systematic benchmark calculations performed under matched conditions are still needed to determine which electrolyte-design principles remain transferable across models and experimental systems. The broader comparison also shows that apparently conflicting trends across the literature often reflect differences in the region of parameter space being explored rather than genuine disagreement between studies. Electrolyte composition, temperature, electrode morphology, surface functionalization, and charging protocol can shift the balance among adsorption, ion packing, screening, and transport. For this reason, conclusions drawn for a particular electrode–electrolyte combination should not be transferred uncritically to another system.

Importantly, these methodological variables are rarely changed independently across independent studies. Differences between reported capacitances therefore cannot generally be assigned uniquely to the force field, electrode charging method, system size, or electrolyte composition. This lack of controlled cross-method comparisons is itself an important limitation of the current literature and explains why apparently different quantitative results should not automatically be interpreted as physical disagreements.

### 3.2 Electrode Curvature, Defects, and Doping

The electrode structures considered in this section are not intended to be an exhaustive survey of graphene engineering strategies. Rather, curvature, defects, and doping are treated as representative test cases in which the electrode structure, electrolyte organization, and simulation methodology become directly coupled. These modifications alter not only ion adsorption and packing at the interface but also, in different ways, the local electronic response of graphene. Consequently, their reliable description may place different demands on the electrode charging and force field treatments introduced in Sections 2 and 2.5.

Beyond pristine graphene, structural engineering provides several independent routes for tuning the performance of graphene-based supercapacitors. Curvature, heteroatom doping, defects, and surface functionalization modify either the electrolyte-side double-layer capacitance or the electrode-side quantum capacitance, or both, often through distinct microscopic mechanisms. Understanding which contribution is affected by each structural modification is therefore essential for rational electrode design.

Curvature is one of the few structural modifications capable of simultaneously influencing both contributions to the total capacitance. Feng et al.^27^ used molecular dynamics simulations to investigate ionic liquids confined at onion-like carbon electrodes of varying radii and found that the double-layer capacitance increases with increasing curvature. The enhancement was attributed to stronger charge overscreening and more efficient ion packing on the convex surfaces. Paek et al.^97^ extended this analysis to carbon nanotube electrodes by combining classical molecular dynamics simulations of the electrolyte with density functional theory calculations of the electrode electronic structure.

Increasing curvature enhanced the double-layer capacitance by reducing the potential drop across the electric double layer through changes in the interfacial ion distribution and radial electric field decay, while the quantum capacitance also increased because smaller-radius nanotubes possess a higher electronic density of states near the Fermi level. However, because the total capacitance is determined by the series combination of the double-layer and quantum contributions, the overall benefit of increasing the curvature depends strongly on the applied potential, with the dominant limiting capacitance shifting between the two regimes. Together, these studies show that curvature simultaneously modifies the electrolyte and electronic responses of graphene-derived electrodes, but the extent to which this translates into improved device performance depends on the operating conditions.

Unlike curvature, which modifies the electrode geometry, heteroatom doping tunes the interfacial capacitance primarily by altering the electronic structure of graphene. Paek et al.^28^ combined density functional theory calculations with classical molecular dynamics simulations of [BMIM]­[PF_6_] at nitrogen-doped graphene to separate the quantum and double-layer contributions to the total capacitance. Two experimentally relevant nitrogen configurations, substitutional and pyridinic nitrogen, were found to substantially increase the quantum capacitance by introducing electronic states near the Fermi level while leaving the electric double-layer microstructure and capacitance virtually unchanged. The enhanced capacitance reported experimentally for nitrogen-doped graphene therefore originates primarily from the electronic response of the electrode rather than from changes in the electrolyte.

More recently, Wang et al.^98^ employed constant-potential molecular dynamics to compare boron-, nitrogen-, and sulfur-doped graphene (see Figure 10), demonstrating that different dopants influence charge storage through distinct microscopic mechanisms. Boron doping directly increased the double-layer capacitance by introducing additional charge-storage sites, whereas nitrogen doping enhanced ionic conductivity by weakening electrode–electrolyte interactions and promoting faster ion transport. These studies collectively show that heteroatom doping should not be regarded as a single design strategy, but rather as a family of chemically distinct modifications capable of selectively targeting different aspects of charge storage and charging dynamics. The importance of tuning the electronic structure is further illustrated by work beyond graphene derivatives. Using joint density-functional-theory calculations, Zhan et al.^99^ predicted that two-dimensional boron sheets (borophene) could exhibit specific capacitances approximately four times larger than graphene, suggesting that future improvements may arise not only from modifying graphene but also from exploring entirely new two-dimensional electrode materials.

**Figure 10:**
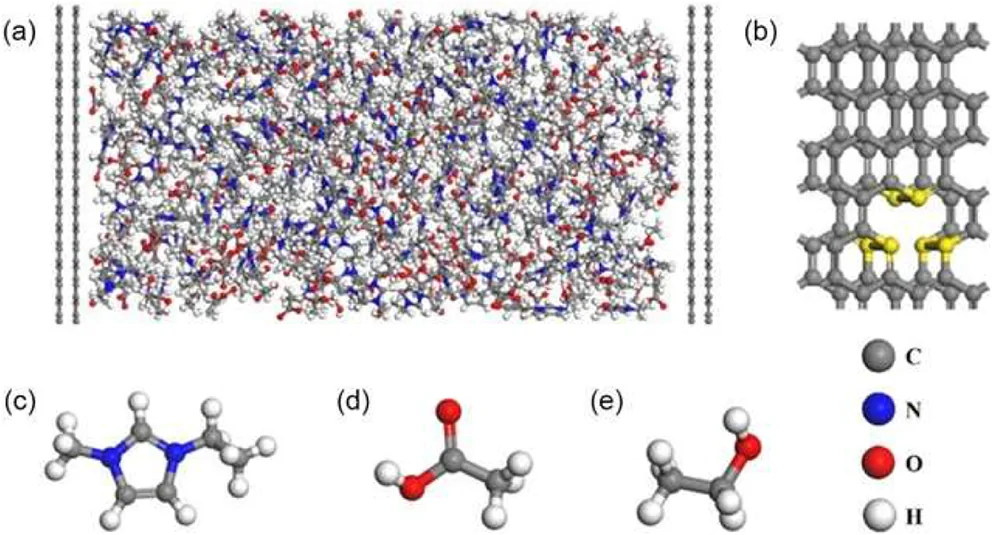
Representative pristine and heteroatom-doped graphene structures employed in molecular dynamics simulations of ionic-liquid supercapacitors. Boron, nitrogen, and sulfur substitution modify the local electronic structure and provide chemically distinct active sites that influence ion adsorption, charge storage, and charging dynamics. Reproduced from Wang et al.^98^ with permission. Copyright 2026 Wiley-VCH GmbH.

These results also illustrate why apparently different conclusions about the effect of doping can arise when different studies monitor different components of the electrochemical response. An increase in total capacitance does not necessarily imply a stronger electric double layer: a structural modification may instead increase the electrode density of states and hence the quantum capacitance, while leaving the interfacial ionic organization nearly unchanged. Conversely, modifications that improve ionic conductivity or create additional interfacial charge-storage sites need not produce the same electronic response. Comparisons among doped graphene electrodes therefore require explicit identification of whether the reported enhancement originates from double-layer capacitance, quantum capacitance, transport kinetics, or a combination of these contributions.

Surface functionalization provides a third route for tailoring electrode performance by modifying the interaction between graphene and the electrolyte without substantially altering the underlying carbon framework. Fileti and Colherinhas^100^ investigated graphene electrodes functionalized with C_60_ fullerenes using molecular dynamics simulations and showed that the fullerene decoration increases the effective electrode surface area by approximately 70%, leading to capacitance enhancements of up to 150% relative to pristine graphene. The improvement arises from the modified electrode–electrolyte interface created by the fullerene layer, which promotes stronger ion adsorption and electric double layer formation. The authors also observed that the intrinsic asymmetry of the ionic liquid limits the overall capacitance because the fullerenes interact preferentially with one ionic species, suggesting that further gains could be achieved by optimizing both the fullerene surface coverage and the electrolyte composition.

The beneficial effect of surface modification is not, however, universal. Constant-potential simulations of graphene oxide electrodes have shown that oxygen-containing functional groups and porous morphologies can substantially reorganize the ionic liquid interface and modify the microscopic charging mechanism.^101^ Direct comparisons between graphitic and oxidized graphene-based surfaces further indicate that oxidation can disrupt the highly ordered ionic layering characteristic of graphitic interfaces and modify overscreening and charge storage.^102^ These results provide an important counterpoint to the capacitance enhancement obtained through C_60_ decoration: surface modification can either improve or impair charge storage depending on whether it increases accessible interfacial area and favorable ion adsorption or instead disrupts ion packing and electrostatic screening.

Among surface modifications, graphene oxide represents perhaps the most technologically relevant departure from pristine graphene. DeYoung et al.^103^ systematically varied the degree of hydroxyl functionalization from pristine graphene to fully oxidized graphene oxide and found that the double-layer capacitance decreases monotonically with increasing oxidation, consistent with experimental observations. The reduction was attributed to two cooperative effects: hydrogen bonding between hydroxyl groups and the electrolyte hinders ion rearrangement, while the oxygen-containing functional groups increase the effective separation between the electrode and screening ions, shifting the electric double layer away from the surface. The detailed oxidation pattern also proved to be important, as different hydroxyl distributions led to different ion accessibilities and capacitances despite identical average oxidation levels. Park et al.^104^ reached similar conclusions for both ionic-liquid and organic electrolytes, showing that oxidation reduces capacitance in each case, although through distinct charge-screening mechanisms.

Beyond its influence on capacitance, oxidation also modifies the microscopic organization of the electrolyte. Using constant-potential molecular dynamics, Dočkal et al.^105^ demonstrated that hydroxyl groups fundamentally reorganize the interfacial hydrogen-bond network, thereby changing the positions of ion adsorption layers and the preferential adsorption of different cations. These effects cannot be explained solely by classical electric-double-layer models but arise from the interplay between direct electrode–solution interactions, hydrogen-bonding networks, and electrostatic charge screening. From the electronic-structure perspective, Pak and Hwang^106^ showed that oxidation also affects the quantum capacitance, revealing that the total interfacial capacitance is optimized at an intermediate degree of hydroxyl functionalization, where the enhancement in quantum capacitance compensates for the simultaneous reduction in double-layer capacitance. Together, these studies show that graphene oxide electrodes must be engineered by balancing electronic and electrochemical effects rather than simply maximizing or minimizing the degree of oxidation.

Architectural design offers yet another route for improving graphene-based supercapacitors without relying on chemical modification of the electrode surface. Chagas et al.^107^ investigated multilayer graphene supercapacitors containing embedded conductive layers within the electrolyte. These internal conductors generate additional electrode–electrolyte interfaces, producing multiple electric double layers throughout the device. Although partitioning the system into electrochemical subsystems reduces the total integral capacitance because the interfaces are effectively connected in series, the accompanying increase in the overall potential difference more than compensates for this loss, yielding volumetric energy density gains exceeding 100% for dual-conductor configurations. The authors therefore identified interface engineering, rather than changes in electrode chemistry, as a promising strategy for increasing the energy storage capability of graphene-based supercapacitors.

A related approach was examined by Bo et al.,^108^ who used molecular dynamics simulations to investigate aqueous electrolytes confined within vertically aligned graphene channels. They found that the electric double-layer structure depends jointly on channel width, surface charge density, and ion size. In narrow channels, ions must partially shed their hydration shells before entering the pores; therefore, charging occurs predominantly through counterion insertion. Wider channels, in contrast, exhibit the more familiar charging mechanism based on counterion adsorption accompanied by co-ion exchange. The optimum channel width was found to lie between the crystal and hydrated ion diameters, demonstrating that three-dimensional electrode architecture provides an additional means of controlling ion accessibility and electric double-layer formation, complementing the surface-engineering strategies discussed above.

Curvature, heteroatom doping, surface functionalization, and architectural design therefore represent complementary rather than competing strategies for engineering graphene-based supercapacitors. Curvature modifies both the electrolyte and the electrode simultaneously, coupling changes in electric double-layer and quantum capacitance. Heteroatom doping primarily tailors the electronic structure of the electrode, whereas surface functionalization reorganizes the electrode–electrolyte interface through specific chemical interactions. Architectural modifications, in turn, regulate ion accessibility, confinement, and the formation of multiple electric double layers without necessarily changing the surface chemistry. Rather than seeking a universally optimal electrode, these studies collectively show that the most effective structural modification depends on the performance metric being targeted, whether maximizing capacitance, enhancing energy density, accelerating ion transport, or improving electrochemical stability.

Although these studies demonstrate that curvature, doping, oxidation, functionalization, and architectural design can each modify charge storage, the available evidence remains fragmented. Most investigations vary one structural feature at a time and frequently evaluate either the electrolyte-side double-layer capacitance or the electrode-side quantum capacitance rather than calculating both contributions self-consistently. Moreover, results obtained for carbon nanotubes, onion-like carbons, borophene, or ideally patterned graphene provide valuable mechanistic insights but cannot necessarily be transferred quantitatively to experimentally synthesized graphene, where curvature, vacancies, edge sites, dopants, and oxygen-containing groups coexist with heterogeneous concentrations and spatial distributions. The reported performance enhancements should therefore be interpreted primarily as design hypotheses rather than as universally additive gains. Establishing their practical relevance will require simulations that combine multiple realistic structural features with electron-structure-aware electrode models and direct experimental validation under matched conditions.

### 3.3 Anomalous Capacitance and Nanoconfinement

Nanoconfinement fundamentally changes the mechanism of charge storage in electrochemical double-layer capacitors. Classical continuum models predict that pores narrower than the solvated ion diameter should contribute little to capacitance because steric constraints prevent efficient ion adsorption. Experiments on carbide-derived carbons, however, revealed precisely the opposite behavior. Chmiola et al.^109^ systematically varied the average pore width between 0.6 and 2.25 nm and demonstrated that the capacitance increases sharply when the pore size approaches the molecular dimensions of the electrolyte, directly challenging the long-standing assumption that pores smaller than solvated ions are electrochemically inaccessible. Largeot et al.^18^ subsequently extended this finding to solvent-free ionic liquids and showed that the maximum capacitance occurs when the pore width closely matches the diameter of the bare, rather than the solvated ion. Remarkably, deviations of only a few tenths of a nanometer from this optimum pore size produced substantial losses in capacitance, highlighting the need to match the pore size to the electrolyte with subnanometer precision. Together, these experiments established nanoconfinement and partial desolvation as the key ingredients underlying the anomalous capacitance.

The microscopic origin of this anomalous behavior was clarified by Kondrat and Kornyshev,^19^ who combined molecular dynamics and Monte Carlo simulations to investigate ionic liquids confined within slit-shaped nanopores. They showed that image-charge screening strongly suppresses the electrostatic repulsion between neighboring counterions, allowing ions of the same charge to pack much more densely than at a planar interface. The resulting “superionic” state is governed primarily by steric packing rather than Coulombic repulsion and provides a microscopic explanation for the experimentally observed capacitance enhancement.

Nanoconfinement does not, however, lead to a single optimum pore size. Feng and Cummings^20^ used molecular dynamics simulations of slit pores to show that capacitance exhibits damped oscillations as the pore width increases. Besides reproducing the anomalous maximum observed experimentally near the bare ion diameter, they predicted a second capacitance maximum and showed that the entire capacitance–pore-size relationship reflects the interference between the electric double layers formed at the opposing pore walls. More generally, the oscillatory behavior tracks the discrete number of ionic layers that can be accommodated within the confined space.

The oscillatory behavior predicted for ideal slit pores is not, however, universal. Using atomistic molecular dynamics simulations that explicitly account for ion shape and electrode polarizability, Wu et al.^110^ showed that the capacitance–pore-size relationship depends sensitively on the solvation structure of the confined electrolyte and its response to electrode charging. Rather than exhibiting a universal oscillatory profile, different ion pairs can display distinct scaling behaviors, reflecting the delicate interplay between pore geometry, ion size, and confinement-induced desolvation. Their work also highlighted that nanoconfined electric double layers constitute a regime in which conventional transmission-line descriptions of porous electrodes cease to be applicable. These key features of the capacitance–pore-size relationship are summarized schematically in Figure 11, which highlights the anomalous maximum near the bare ion diameter and the subsequent damped oscillations expected under nanoconfinement.

**Figure 11:**
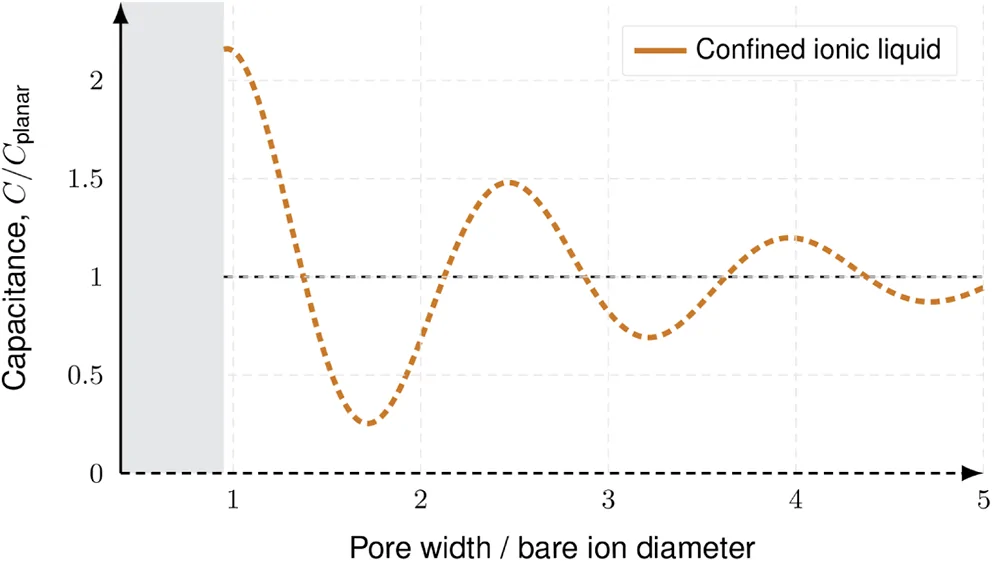
Schematic capacitance as a function of pore width, normalized by the bare ion diameter, relative to the planar (unconfined) limit. Pores narrower than the bare ion are sterically inaccessible (shaded region). Just above this threshold, capacitance rises to an anomalous maximum, the Largeot et al.^18^ and Chmiola et al.^109^ results discussed in Section 1, and oscillates with decaying amplitude as the pore widens further, tracking the number of discrete ion layers that fit within the pore, before converging to the planar limit at large pore width. The curve is illustrative, not extracted from a specific simulation; the oscillation is not universal across ionic liquids and pore geometries.^110^

Merlet et al.^111^ subsequently demonstrated that the microscopic mechanisms identified in idealized slit pores also govern charge storage in realistic disordered nanoporous carbons. Combining molecular dynamics simulations with experimental measurements, they showed that the enhanced capacitance arises not simply from the large internal surface area, but from the confinement-induced separation of counterions and co-ions within the pore network. Charging proceeds predominantly through ion exchange with the bulk electrolyte, while the amount of liquid inside the pores remains nearly constant, accompanied by partial desolvation and suppression of overscreening. The resulting capacitances were in quantitative agreement with experiments, establishing confinement-induced ion packing as the microscopic origin of the anomalous capacitance observed in nanoporous carbon electrodes.

The apparent differences among these confinement studies, therefore, reflect, at least in part, different levels of structural idealization. Ideal slit-pore models are particularly useful for isolating ion-size and pore-width effects and reveal discrete layering and oscillatory capacitance with exceptional clarity, whereas atomistically realistic or disordered porous electrodes introduce distributions of pore sizes, connectivity, and local environments that broaden or suppress these idealized signatures. Accordingly, the oscillatory capacitance predicted for a uniform slit pore should be interpreted as a mechanistic limiting case rather than as a universal capacitance–pore-size law for realistic carbon electrodes.

For graphene-based electrodes, nanoconfinement influences not only the electric double layer but also the electronic response of the electrode. Gameel et al.^112^ developed a first-principles model of graphene slit pores containing highly confined ionic liquid ions, allowing the quantum capacitance of pore/ion systems to be evaluated explicitly. Their calculations showed that the quantum capacitance depends not only on the pore geometry but also on the identity of the confined ion, the applied potential, and the presence of nitrogen dopants. Whereas confinement alone produces only minor changes near the potential of zero charge, both ion insertion and pore doping substantially modify the electronic response at higher potentials, demonstrating that quantum capacitance under extreme confinement emerges from the coupled electronic structures of the graphene pore and the confined electrolyte rather than from the electrode alone. This result extends the discussion of Section 2.3 to nanoporous systems, showing that the electrode and electrolyte become electronically intertwined under extreme confinement.

The broader implications of this coupling were highlighted by Nesterova et al.,^33^ who reviewed recent theoretical and molecular simulation studies of ionic liquids under nanoconfinement. They concluded that the structural, chemical, and electronic properties of the confining material play a far more important role than traditionally assumed, yet remain insufficiently represented in existing theoretical descriptions. Their analysis emphasized that future progress will require molecular models that treat the electrolyte and confining surface as a coupled system, incorporating realistic surface morphology, chemical functionalization, and an electronic response to accurately describe ionic liquids in confinement.

The nanoconfinement literature provides compelling evidence that ion packing, partial desolvation, image-charge screening, and suppression of overscreening can enhance capacitance, but the quantitative transferability of these mechanisms to graphene-based devices remains uncertain. Much of the strongest experimental validation concerns carbide-derived or disordered nanoporous carbons, whereas graphene-specific studies frequently employ ideal slit pores, uniform pore widths, or first-principles models that are necessarily limited to small systems. The often-cited condition that capacitance is maximized when the pore width approaches the bare-ion diameter should therefore not be treated as a universal design rule because the optimum depends on ion shape, solvation, electrode polarizability, surface chemistry, and the distribution and connectivity of accessible pores. In addition, the equilibrium capacitance alone does not reveal whether strongly confined ions can enter and leave the pore network rapidly enough for practical charging. Predictive studies should consequently examine capacitance, ion accessibility, and charging kinetics together in structurally disordered graphene pores with dynamically responsive electrodes.

### 3.4 Machine-Learning Interatomic Potentials: The Current Frontier

Throughout the previous sections, a recurring compromise emerged. First-principles methods accurately describe electronic structure and electrode polarization but remain restricted to relatively small systems and short simulation times, whereas classical force fields enable nanosecond simulations of realistic supercapacitors at the cost of introducing simplified analytical interaction models and system-specific parametrization. Machine-learning interatomic potentials (MLIPs) have recently emerged as a promising strategy to bridge this gap by learning the potential energy surface directly from quantum-mechanical reference data, combining near-DFT accuracy with computational efficiency comparable to that of classical molecular dynamics.^113^ Figure 12 summarizes the resulting workflow.

**Figure 12:**
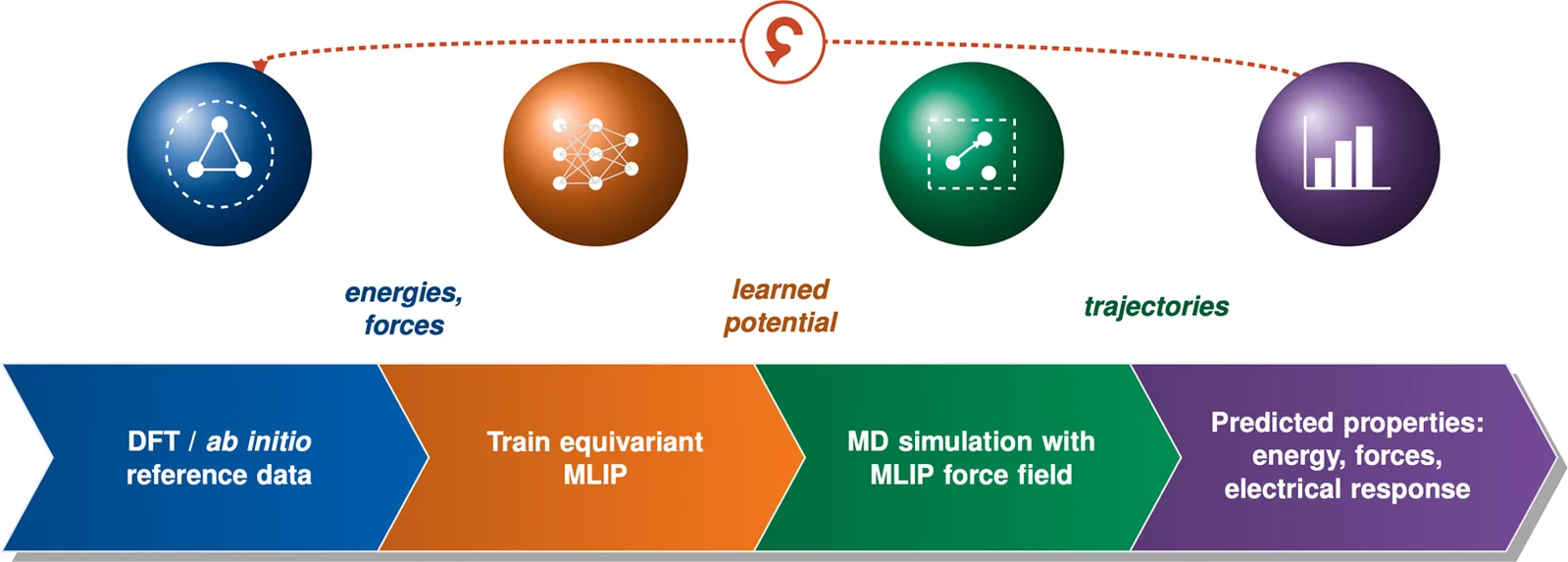
Schematic MLIP workflow. A quantum-chemistry reference data set (typically energies and forces from DFT calculations) is used to train a machine-learning model capable of reproducing the underlying potential-energy surface. The resulting potential is then employed in molecular dynamics simulations to predict structural, dynamical, and electrochemical properties with substantially lower computational cost than direct first-principles calculations. The dashed arrow denotes the active-learning loop, in which configurations sampled during MD are recomputed at the reference level and added to the training set. Electrical response is not a direct output of potentials trained only on energies and forces; it requires response-augmented training targets or long-range MLIP architectures (Section 3.4).

Despite the rapid growth of machine learning in electrochemical materials research, studies that directly combine ML and molecular dynamics for graphene-based supercapacitors containing ionic liquid electrolytes remain comparatively scarce. Nevertheless, recent work in closely related systems demonstrates several routes through which ML can address limitations that are directly relevant to this field.

One particularly promising direction is the development of machine-learning force fields that approach first-principles accuracy while retaining the system sizes and time scales accessible to classical MD. Ling et al.^114^ constructed deep-learning force fields for imidazolium-based ionic liquids with ten different anions using training data generated from *ab initio* molecular dynamics. The resulting models reproduced energies, forces, structural properties, vibrational behavior, and intermolecular interactions with accuracy close to the underlying first-principles description while enabling nanosecond-scale MD simulations. Such approaches are particularly attractive for supercapacitor simulations because they could provide a route between computationally inexpensive empirical force fields and the much greater computational cost of explicitly electronic-structure-based dynamics.

The feasibility of this strategy for electrochemical interfaces was already demonstrated in carbon-based supercapacitors. Zhang et al.^115^ employed machine learning-accelerated molecular dynamics (MLMD) to investigate electrified microporous carbon/electrolyte interfaces at different electrode potentials. The machine-learning force field was trained from AIMD data and enabled simulations extending to the nanosecond regime, allowing the authors to resolve ion and solvent distributions, ion insertion into micropores, and the dependence of charge storage on the pore size and electrode potential. This example is particularly relevant because it shows that MLMD can extend the limited sampling accessible to AIMD while retaining an electronic-structure-informed description of the electrochemical interface.

This approach was subsequently extended to chemically modified carbon electrodes. Zhang et al.^116^ combined AIMD and MLMD to investigate nitrogen-doped planar and microporous carbon interfaces, showing that the effect of nitrogen functionality depends strongly on the electrode dimensionality and confinement. In particular, the longer MLMD trajectories allowed differences in electrolyte transport and ion storage within doped micropores to be resolved that were not apparent from planar electrode models alone. These results illustrate how machine-learning potentials can be used not only to accelerate sampling but also to examine the coupling between electrode chemistry, pore structure, and electrolyte dynamics.

For ionic liquids, the principal challenge extends beyond reproducing a single electrolyte: practical applications often involve mixtures, additives, or entirely new ionic species outside the training set. Goodwin et al.^117^ addressed this problem by using equivariant MLIPs trained on a diverse set of imidazolium- and pyrrolidinium-based ionic liquids. Rather than developing an individual potential for each electrolyte, they demonstrated compositional transferability by accurately predicting the properties of ionic-liquid mixtures excluded from the training data set. They further validated this approach against a newly synthesized ionic liquid, reproducing experimental densities, thermal expansion coefficients, and compressibilities using a model trained with only approximately 200 DFT configurations. These results suggest that MLIPs can substantially reduce the effort required to parametrize atomistic models while retaining predictive accuracy across chemically related electrolyte families.

Extending MLIPs from bulk electrolytes to electrochemical interfaces is considerably more challenging because the interaction potential depends explicitly on the applied electrode potential. Conventional MLIPs are typically trained on neutral configurations and therefore lack an explicit description of electrical response. Bergmann et al.^118^ addressed this limitation by introducing a response-augmented framework in which the work function and its dependence on applied bias are learned alongside the interatomic potential. Using Born effective charges to stabilize the learning process, their approach enables finite-bias molecular dynamics while preserving the computational efficiency of standard MLIPs, providing a practical route toward simulations of electrified solid–liquid interfaces. Shortly afterward, Zhong et al.^119^ demonstrated that electrical-response properties such as polarization, Born effective charges, infrared spectra, and ionic conductivity can, in some cases, be recovered directly from long-range MLIPs trained only on energies and forces, without explicitly including polarization or charge information during training. Together, these studies indicate that MLIPs are beginning to capture not only atomic interactions but also the electromechanical response that governs electrochemical systems.

These developments form part of a broader transition in computational electrochemistry reviewed recently by Jinnouchi and Minami.^113^ Their analysis highlights how MLIPs are rapidly evolving from accurate surrogate potentials into general multiscale modeling tools capable of predicting transport coefficients, free energy landscapes, reaction kinetics, and materials properties across length and time scales inaccessible to conventional first-principles calculations. Rather than replacing electronic structure methods, MLIPs increasingly complement them by preserving quantum-level accuracy while enabling statistically meaningful molecular simulations.

A complementary application of ML is the high-throughput screening of ionic-liquid electrolytes. Manna et al.^120^ combined MD–DFT calculations with supervised machine learning to predict the electrochemical windows of ionic liquids. Their model was subsequently used to screen 660 ionic-liquid electrolytes, demonstrating how molecular simulations can provide physically grounded training data while ML expands the searchable chemical space far beyond that accessible through individual MD or DFT calculations. Although this study targeted dual-ion batteries rather than supercapacitors, the underlying strategy is directly relevant to ionic-liquid-based supercapacitors, for which electrochemical stability and operating voltage are central determinants of the achievable energy density.

Despite this rapid progress, no MLIP has yet been demonstrated for a fully realistic graphene/ionic liquid supercapacitor combining constant-potential electrodes, electronic polarization, quantum capacitance, and machine-learned interatomic interactions within a single computational framework. Bridging these ingredients remains one of the major methodological challenges for atomistic simulations of electrochemical energy storage. In a recent Perspective on sodium-ion capacitors, Fan et al.^121^ argued that overcoming the remaining limitations of atomistic simulations will require the integration of physics-informed machine learning, multiscale modeling, and data-driven materials discovery into unified computational workflows. Although their discussion focused on sodium-ion capacitors, the same conclusion applies naturally to graphene-based supercapacitors, where the combination of accurate electronic structure descriptions, realistic electrolyte dynamics, and transferable MLIPs appears to be the next frontier for predictive molecular simulation.

Therefore, these developments suggest three complementary roles for machine learning in future molecular simulations of graphene/ionic-liquid supercapacitors: (i) ML force fields can extend electronic-structure-level descriptions toward the length and time scales required for equilibrating complex electric double layers; (ii) ML-accelerated MD can enable systematic exploration of electrode chemistry, confinement, and potential-dependent charging mechanisms; and (iii) surrogate models trained on MD and electronic-structure data can screen large libraries of ionic liquids before more computationally demanding interfacial simulations are performed. A particularly promising future workflow would therefore combine high-throughput ML screening of electrolyte candidates with ML-driven atomistic simulations of the most promising electrode–electrolyte combinations. Integrating these three capabilities within a unified workflow could transform ML from an acceleration tool into a predictive platform for the rational design of graphene-based/ionic-liquid supercapacitors.

The promise of MLIPs should nevertheless be distinguished from their demonstrated predictive capability. Agreement with first-principles data within a training set does not ensure transferability to electrode potentials, ion compositions, defect structures, or confinement regimes that were not adequately represented during training. Charged graphene/ionic liquid interfaces pose a particularly demanding extrapolation problem because the relevant interactions include long-range electrostatics, potential-dependent charge redistribution, electronic polarization, and structural environments that evolve during charging. A model may therefore reproduce energies and forces while still yielding inaccurate charge distributions, capacitances, or charging kinetics. Future applications should report the chemical and electrochemical domains covered by the training data, validation on configurations excluded from training, and uncertainty estimates during production simulations. Until such evidence is available, MLIPs should be presented as promising enabling models rather than as established substitutes for electronic-structure-aware simulations.

Table 2 lists these studies individually, but a few of their quantitative findings are striking enough to warrant a visual summary of their own. Figure 13 collects eight such results, spanning interfacial capacitance, surface functionalization, cosolvent and green-electrolyte design, electrode architecture, graphyne-vs-graphene comparisons, ion dynamics, and nanoconfinement, arranged around the graphene/ionic- liquid supercapacitor motif that unifies them.

**Figure 13:**
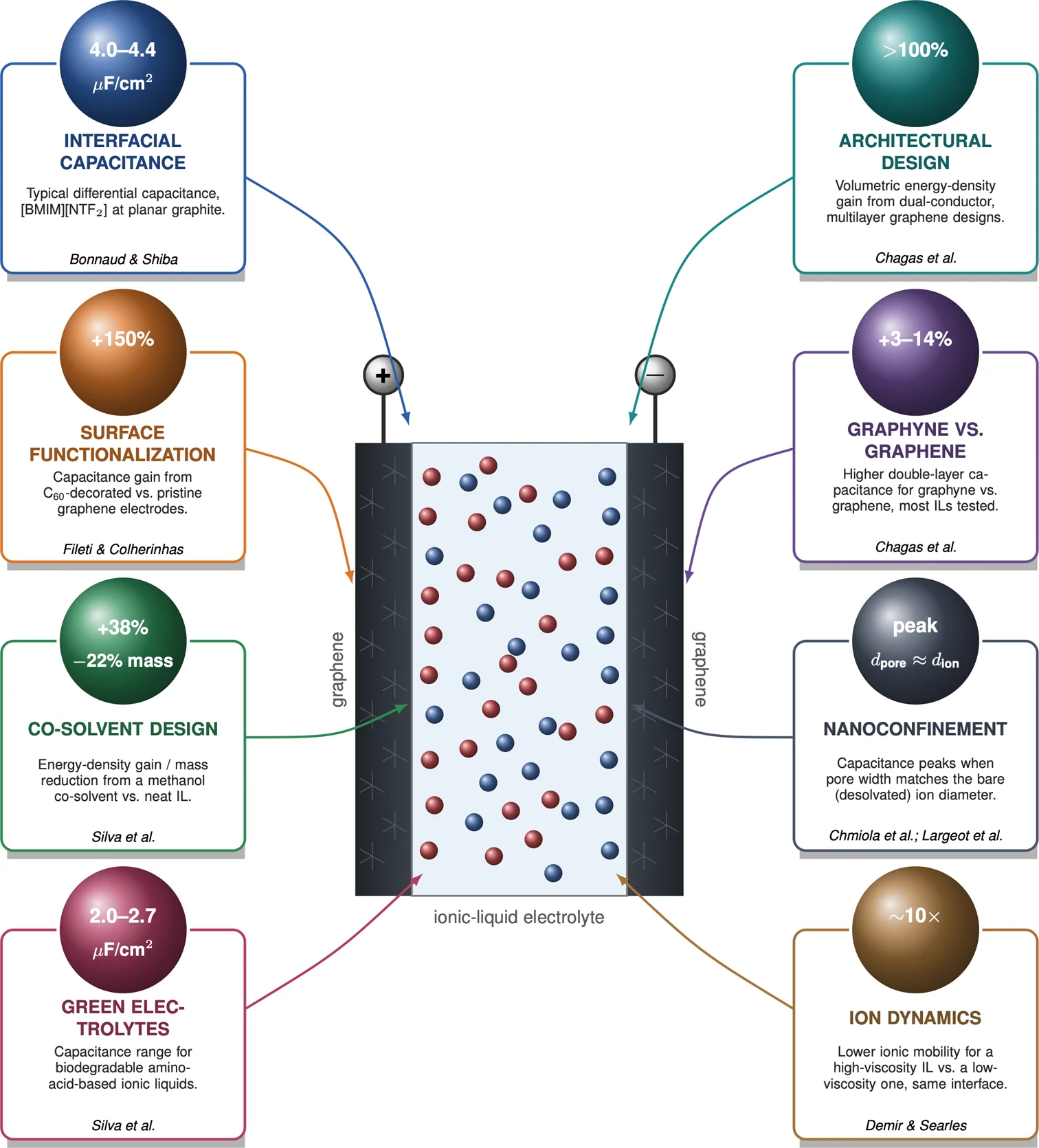
Eight quantitative highlights from the graphene/ionic-liquid supercapacitor studies discussed in this review arranged around a schematic of the interface (cations, blue; anions, red): baseline interfacial capacitance,^73^ surface functionalization,^100^ cosolvent design,^76^ green electrolytes,^78^ electrode architecture,^107^ graphyne vs graphene,^35^ ion dynamics,^74^ and nanoconfinement.^18,109^ These results come from different electrodes, electrolytes, and methods and are not directly comparable to one another; each illustrates a distinct lever for improving graphene-based supercapacitors. Table 2 lists the full set of studies and conditions.

## 4 Current Challenges

Four methodological gaps stand out from the literature review above. First, force-field polarizability and electronic-structure-aware electrode-charging methods have rarely been combined in the same simulation of a graphene/ionic-liquid interface. Bacon et al.^61^ showed that mutual polarization of electrode and electrolyte is necessary for quantitative capacitance agreement in a nanoporous carbon system, but the equivalent combination for graphene specifically, a polarizable ionic-liquid force field paired with a quantum-capacitance-aware CPM treatment of the electrode, has not yet been reported.

Second, a validation gap remains between simulation and experiment. Most direct experimental probes of the graphene/ionic-liquid interface (Section 3.1) report structural information or qualitative capacitance trends rather than the differential-capacitance curves needed for a direct, quantitative comparison against molecular dynamics predictions; closing this gap would require experimental and computational groups to report results for matched electrode potentials, ion concentrations, and temperatures.

Third, curvature, defects, and doping (Section 3.2) have so far been studied one variable at a time. A real graphene electrode combines several of these features simultaneously. Wrinkling and curvature may arise from restacking; edge and vacancy defects are introduced during synthesis; and residual functional groups can produce unintentional doping. How these effects combine (additively, synergistically, or antagonistically) remains largely unexplored.

Fourth, machine-learning interatomic potentials (Section 3.4) have not yet been applied to a charged graphene/ionic liquid interface at all, let alone combined with an electronic-structure-aware treatment of the electrode. Closing this specific gap, rather than developing yet another hand-parametrized polarizable force field, could constitute one of the most consequential methodological advances in the near term.

## 5 Summary and Outlook

This review has organized molecular dynamics simulations of graphene-derived/ionic-liquid supercapacitors around a single argument: that the methods available to the simulator—fixed and constant charge, constant potential, electronic-structure-aware variants, and externally applied fields (Section 2)—together with the choice of force field (Section 2.5), form a toolbox of complementary options rather than a hierarchy of increasing accuracy. Section 2.6 made this comparison explicit, and the results reviewed in Section 3 illustrate why the distinction matters in practice: studies of interfacial structure, electrode curvature and doping, and nanoconfinement each rely on a different combination of methods, chosen for the specific physical quantity under investigation rather than for a single method’s general superiority. Figure 14 summarizes this argument visually, positioning each family of studies within the two-dimensional design space defined by electrode charging and force-field sophistication, and marking the corner of that space that machine-learning interatomic potentials aim to make practically accessible.

**Figure 14:**
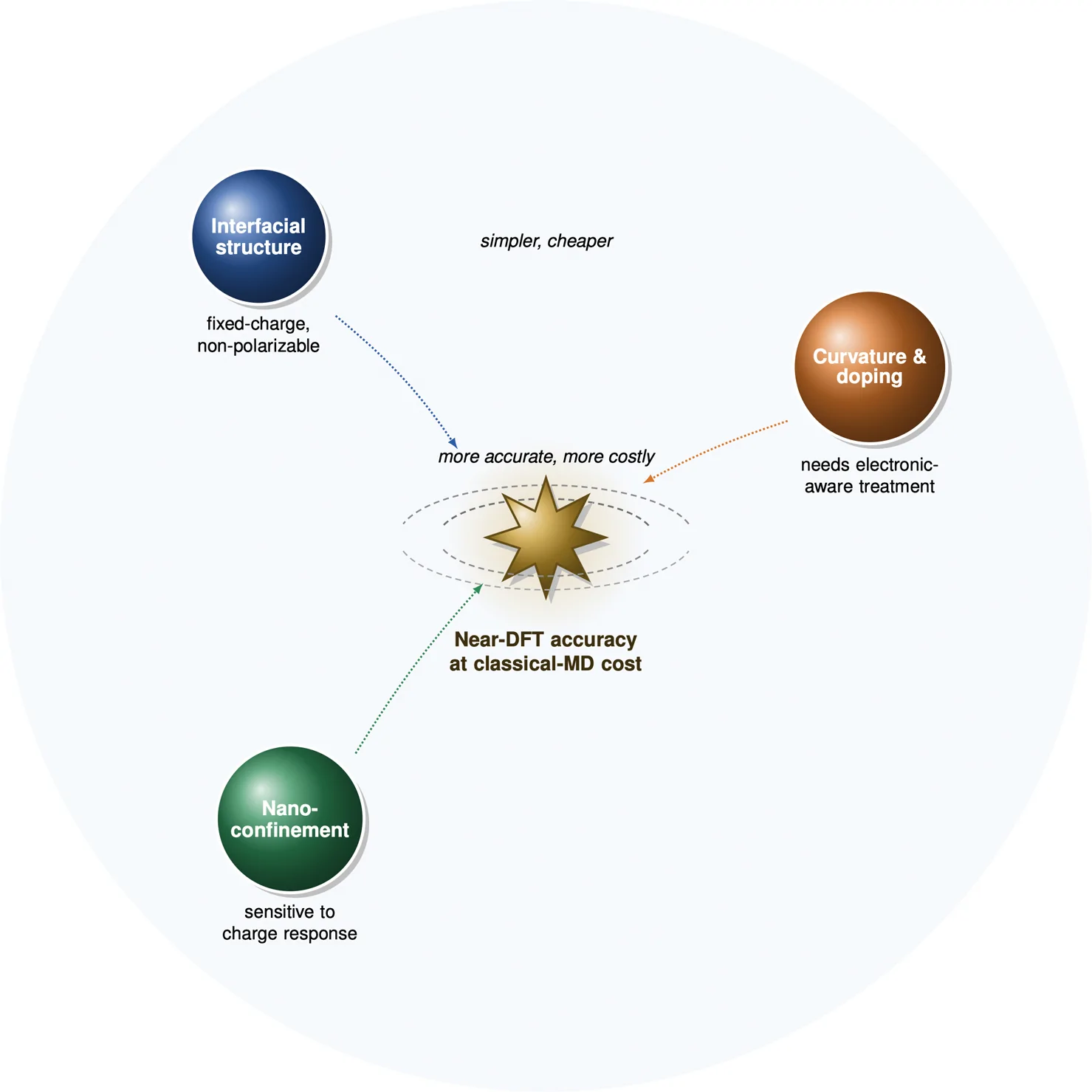
The MD toolbox for graphene-derived/ionic-liquid supercapacitors, shown as distance from an aspirational target rather than as a literal coordinate system. The central star represents the combination that machine learning interatomic potentials aim to make practically accessible: near-DFT accuracy in both the electrode treatment and the force field, at the computational cost of classical MD. Each colored sphere marks, qualitatively, how far a family of studies discussed in Section 3 currently sits from that target, given the combination of electrode-charging method and force-field sophistication it typically relies on: interfacial-structure studies rely on the least expensive, most mature combinations; curvature, defect, and doping studies require an electronic-aware treatment specific to graphene; nanoconfinement studies are especially sensitive to the electrode’s charge response. Dotted arrows indicate the direction, not the distance, in which each area is expected to move as machine-learned potentials mature.

Graphene’s semimetallic band structure adds a dimension to this toolbox that most other carbon electrodes do not require: a non-negligible quantum capacitance that depends on exactly the same structural features—curvature, defects, doping, and confinement—that are also used to tune the double-layer response (Sections 3.2 and 3.3). Reliable graphene-based supercapacitor design, therefore, depends on treating both contributions with comparable fidelity, which, in turn, depends on the continued development of the electronic-structure-aware methods introduced in Section 2.3.

Closing the four gaps identified in Section 4 will require coordinated methodological and experimental advances, but these gaps can also be formulated as a set of concrete questions for the field. How should competing simulation methods be benchmarked under equivalent conditions? Which experimental observables are the most useful for validating molecular models? How much uncertainty arises from the force field choice, electrode representation, finite-size effects, and sampling? How can molecular-scale information be transferred to the larger length and time scales relevant to real devices? How can machine learning be incorporated into this hierarchy without sacrificing physical interpretability and validation?

A first priority is therefore the establishment of common benchmark problems. Comparisons among FCM, CPM, polarizable force fields, electronic structure-aware approaches, and emerging MLIPs are frequently performed for different electrolytes, electrode geometries, cell dimensions, and thermodynamic conditions, making it difficult to isolate the origin of discrepancies between studies. A useful benchmark could consist of a small set of well-characterized graphene/ionic liquid interfaces simulated with different charging methods and force fields while keeping the remaining conditions fixed. Such comparisons should extend beyond integral capacitance to include differential capacitance, ion density and potential profiles, electrode charge distributions, molecular orientation, and charging dynamics. This would provide a direct measure of the uncertainty introduced by the computational model itself and help to establish the regimes in which simpler methods remain reliable.

Experimental validation is a second priority. Direct validation requires simulations and experiments conducted under conditions matched as closely as possible in terms of temperature, electrolyte composition, electrode structure, and applied potential. Capacitance alone, however, is not sufficient to discriminate among molecular models: two methods may reproduce similar macroscopic capacitances while predicting different ion distributions or charging mechanisms. Future validation should therefore combine electrochemical measurements with observables that probe the interfacial structure and dynamics more directly. The experimentally observed layering of ionic liquids at graphene and graphite interfaces already provides an important qualitative test of molecular simulations; extending such comparisons to quantitative, potential-dependent structural and dynamical observables would provide substantially stronger constraints on force fields and electrode models.

Closely related to benchmarking is the need for systematic uncertainty quantification. Statistical error from finite trajectories represents only one component of the uncertainty in an MD prediction. Force field parametrization, electrode-charging method, finite system dimensions, electrode thickness, electrostatic boundary conditions, and equilibration can all influence the calculated interfacial structure and capacitance. Future studies should therefore complement statistical error estimates with sensitivity analyses in which these modeling choices are varied individually, whenever computationally feasible. Such an approach would help distinguish statistical uncertainty from model uncertainty and determine whether reported differences between electrode or electrolyte designs are larger than the uncertainty introduced by the simulation protocol.

A third opportunity lies in the multiscale modeling. Atomistic MD resolves the adsorption, desorption, orientation, and collective rearrangement of ions within the electric double layer, but the dimensions and charging times of realistic porous electrodes remain beyond routine atomistic simulations. A natural hierarchy would connect electronic-structure calculations to atomistic MD, atomistic MD to coarse-grained or mesoscale descriptions, and these models ultimately to continuum or device-scale simulations. Electronic-structure calculations can provide reference interactions and electrode-response properties; atomistic simulations can supply interfacial capacitances, transport coefficients, free-energy information, and microscopic charging mechanisms; and larger-scale models can propagate these quantities through realistic pore networks and electrode architectures. The central challenge is therefore not simply to reach larger scales but to determine which molecular observables can be coarse-grained without losing the interfacial physics responsible for charge storage.

Machine learning provides a possible bridge across several of these challenges. As discussed in Section 3.4, ML force fields have already been developed for ionic liquids, ML-accelerated dynamics has been applied to electrified carbon/electrolyte interfaces, and data-driven models have been used to screen large ionic-liquid chemical spaces. A sufficiently expressive and transferable MLIP could potentially represent electrolyte polarization and electrode response within a unified framework while extending simulations to larger nanoconfined systems and longer charging trajectories. The outstanding challenge is to combine these capabilities with potential-dependent electrode response, quantum capacitance, and rigorous uncertainty estimation. A promising workflow would use electronic-structure calculations to generate reference data, MLIPs to extend atomistic sampling, surrogate models to screen electrode–electrolyte combinations, and targeted experiments to validate the resulting predictions.

Ultimately, progress toward predictive simulation will depend not on increasing methodological complexity alone, but on demonstrating that predictions are transferable, experimentally testable, and accompanied by meaningful estimates of uncertainty. Common benchmark systems, matched simulation–experiment data sets, systematic sensitivity analyses, and multiscale workflows connecting electronic structure, molecular dynamics, machine learning, and device-level modeling would provide the foundation for this transition. Such integration would move molecular simulation beyond explaining individual trends and toward predicting which combinations of ionic-liquid chemistry and graphene-derived electrode structure should be prioritized for experimental synthesis and testing.

## 6 Conclusions

Graphene and graphene-derived ionic-liquid supercapacitors provide a particularly well-defined platform for examining how electrode response, electrolyte organization, and nanoconfinement jointly determine interfacial charge storage. The literature reviewed here shows that reliable molecular simulation does not depend on identifying a universally superior electrode model or force field, but on matching the methodological resolution to the physical observable of interest. Fixed-charge, constant-potential, electronic-structure-aware, field-based, polarizable, and coarse-grained approaches therefore remain complementary, provided that their assumptions and limitations are made explicit.

The next decisive step is integration rather than the continued isolation of individual effects. Mutual electrode–electrolyte polarization, quantum-capacitance-aware charge response, realistic combinations of curvature, defects, doping, and functionalization, and transferable machine-learning interatomic potentials must be examined within common frameworks anchored in experimental evidence. Achieving this integration would move molecular dynamics beyond the explanation of individual trends and toward the predictive design of graphene-based supercapacitors.
